# Disease stage–specific atrophy markers in Alzheimer's disease

**DOI:** 10.1002/alz.70482

**Published:** 2025-07-21

**Authors:** Hannah Baumeister, Helena M. Gellersen, Sarah E. Polk, René Lattmann, Anika Wuestefeld, Laura E. M. Wisse, Trevor Glenn, Renat Yakupov, Melina Stark, Luca Kleineidam, Sandra Roeske, Barbara Marcos Morgado, Hermann Esselmann, Frederic Brosseron, Alfredo Ramirez, Falk Lüsebrink, Matthis Synofzik, Björn H. Schott, Matthias C. Schmid, Stefan Hetzer, Peter Dechent, Klaus Scheffler, Michael Ewers, Julian Hellmann‐Regen, Ersin Ersözlü, Eike Spruth, Maria Gemenetzi, Klaus Fliessbach, Claudia Bartels, Ayda Rostamzadeh, Wenzel Glanz, Enise I. Incesoy, Daniel Janowitz, Boris‐Stephan Rauchmann, Ingo Kilimann, Sebastian Sodenkamp, Marie Coenjaerts, Annika Spottke, Oliver Peters, Josef Priller, Anja Schneider, Jens Wiltfang, Katharina Buerger, Robert Perneczky, Stefan Teipel, Christoph Laske, Michael Wagner, Gabriel Ziegler, Frank Jessen, Emrah Düzel, David Berron, for the DELCODE study group

**Affiliations:** ^1^ German Center for Neurodegenerative Diseases (DZNE) Magdeburg Germany; ^2^ Cognition and Brain Sciences Unit, University of Cambridge Cambridge UK; ^3^ Department of Psychology University of Cambridge Cambridge UK; ^4^ Institute of Cognitive Neurology and Dementia Research (IKND) Otto‐von‐Guericke University Magdeburg Germany; ^5^ Clinical Memory Research Unit Department of Clinical Sciences Malmö Lund University Lund Sweden; ^6^ Diagnostic Radiology, Department of Clinical Sciences Lund Lund University Lund Sweden; ^7^ Penn Image Computing and Science Laboratory (PICSL) Department of Radiology University of Pennsylvania Philadelphia Pennsylvania USA; ^8^ German Center for Neurodegenerative Diseases (DZNE) Bonn Germany; ^9^ Department for Cognitive Disorders and Old Age Psychiatry University Hospital Bonn Bonn Germany; ^10^ Department of Psychiatry and Psychotherapy University Medical Center Goettingen Goettingen Germany; ^11^ Excellence Cluster on Cellular Stress Responses in Aging‐Associated Diseases (CECAD) Cologne Germany; ^12^ Division of Neurogenetics and Molecular Psychiatry Department of Psychiatry and Psychotherapy University of Cologne Cologne Germany; ^13^ Department of Psychiatry & Glenn Biggs Institute for Alzheimer's and Neurodegenerative Diseases San Antonio Texas USA; ^14^ German Center for Neurodegenerative Diseases (DZNE) Tübingen Germany; ^15^ Division of Translational Genomics of Neurodegenerative Diseases Hertie Institute for Clinical Brain Research Tübingen Germany; ^16^ Center for Neurology University of Tübingen Tübingen Germany; ^17^ German Center for Neurodegenerative Diseases (DZNE) Goettingen Germany; ^18^ Leibniz Institute for Neurobiology Magdeburg Germany; ^19^ Institute for Medical Biometry Informatics and Epidemiology, University Hospital Bonn Bonn Germany; ^20^ Berlin Center for Advanced Neuroimaging Charité – Universitätsmedizin Berlin Berlin Germany; ^21^ MR‐Research in Neurosciences, Department of Cognitive Neurology University Medical Center Goettingen Goettingen Germany; ^22^ Department for Biomedical Magnetic Resonance University of Tübingen Tübingen Germany; ^23^ German Center for Neurodegenerative Diseases (DZNE) Munich Germany; ^24^ Institute for Stroke and Dementia Research (ISD) University Hospital LMU Munich Munich Germany; ^25^ German Center for Neurodegenerative Diseases (DZNE) Berlin Germany; ^26^ Department of Psychiatry and Neurosciences Charité – Universitätsmedizin Berlin Berlin Germany; ^27^ ECRC Experimental and Clinical Research Center Charité – Universitätsmedizin Berlin Berlin Germany; ^28^ Institute of Psychiatry and Psychotherapy Charité – Universitätsmedizin Berlin Berlin Germany; ^29^ Department of Psychiatry University of Cologne Cologne Germany; ^30^ Department of Psychiatry and Psychotherapy University Clinic Magdeburg Otto‐von‐Guericke University Magdeburg Germany; ^31^ Department of Psychiatry and Psychotherapy University Hospital, LMU Munich Munich Germany; ^32^ Sheffield Institute for Translational Neuroscience (SITraN) University of Sheffield Sheffield UK; ^33^ Department of Neuroradiology University Hospital LMU Munich Munich Germany; ^34^ German Center for Neurodegenerative Diseases (DZNE) Rostock Germany; ^35^ Department of Psychosomatic Medicine Rostock University Medical Center Rostock Germany; ^36^ Department of Psychiatry and Psychotherapy University of Tübingen Tübingen Germany; ^37^ Department of Neurology University Hospital Bonn Bonn Germany; ^38^ University of Edinburgh and UK DRI Edinburgh UK; ^39^ Department of Psychiatry and Psychotherapy School of Medicine and Health Technical University of Munich Munich Germany; ^40^ German Center for Mental Health (DZPG) Munich Germany; ^41^ Neurosciences and Signaling Group Institute of Biomedicine (iBiMED) Department of Medical Sciences University of Aveiro Aveiro Portugal; ^42^ Munich Cluster for Systems Neurology (SyNergy) Munich Germany; ^43^ Ageing Epidemiology Research Unit (AGE) School of Public Health Imperial College London London UK; ^44^ Section for Dementia Research, Hertie Institute for Clinical Brain Research Tübingen Germany; ^45^ Center for Behavioral Brain Sciences (CBBS) Otto‐von‐Guericke University Magdeburg Germany

**Keywords:** imaging biomarker, longitudinal atrophy, magnetic resonance imaging, medial temporal lobe, parietal lobe

## Abstract

**INTRODUCTION:**

Structural magnetic resonance imaging (MRI) often lacks diagnostic, prognostic, and monitoring value in Alzheimer's disease (AD), particularly in early disease stages. To improve its utility, we aimed to identify optimal atrophy markers for different intended uses.

**METHODS:**

We included 363 older adults; cognitively unimpaired individuals who were negative or positive for amyloid beta (Aβ) and Aβ‐positive patients with subjective cognitive decline, mild cognitive impairment, or dementia of the Alzheimer type. MRI and neuropsychological assessments were administered annually for up to 3 years.

**RESULTS:**

Accelerated atrophy of medial temporal lobe subregions was evident already during preclinical AD. Symptomatic disease stages most notably differed in their hippocampal and parietal atrophy signatures. Atrophy–cognition relationships varied by intended use and disease stage.

**DISCUSSION:**

With the appropriate marker, MRI can detect abnormal atrophy already during preclinical AD. To optimize performance, atrophy markers should be tailored to the targeted disease stage and intended use.

**Highlights:**

- Subregional atrophy markers detect ongoing atrophy in preclinical Alzheimer's disease (AD).
- Subjective cognitive decline in preclinical AD links to manifest atrophy.
- Optimal atrophy markers differ by the disease stage and intended use.

## 1 BACKGROUND

Aside from accumulations of amyloid beta (Aβ) and neurofibrillary tangles (NFTs) of hyperphosphorylated tau, neurodegeneration is a pathological hallmark of Alzheimer's disease (AD).^1^ Structural magnetic resonance imaging (MRI) is commonly used for assessing gray matter atrophy as a downstream biomarker of neurodegeneration, offering advantages like non‐invasiveness, good accessibility, and robust associations with clinical phenotypes.^2^, ^3^ MRI‐based atrophy assessments typically rely on quantitative measures of gray matter volume or cortical thickness, either globally or in regions of interest (ROIs), including the hippocampus or the “AD signature cortex.” While often applied across disease stages, these generic markers tend to lack sensitivity to subtle changes in early disease stages, particularly compared to fluid biomarkers or molecular imaging.^1^, ^4^, ^5^, ^6^ This limitation is critical, as preclinical and early prodromal disease stages are increasingly recognized as key windows for effective disease‐modifying intervention.^7^, ^8^

Atrophy assessment at a given AD stage may be improved by guiding marker selection by the spatiotemporal progression of underlying pathology. Previous studies have demonstrated that NFTs tend to co‐localize with subsequent hotspots of neurodegeneration.^3^, ^9^, ^10^, ^11^ The medial temporal lobe (MTL) is among the earliest brain regions affected by AD‐related NFTs and its subregions—including hippocampal subfields, the amygdala, and cortical structures—exhibit varying degrees and timelines of NFT accumulation.^10^, ^12^, ^13^, ^14^, ^15^ This makes MTL subregional volumetry a promising approach for providing fine‐grained insights into early AD progression. In fact, previous research has shown that MTL subregions particularly vulnerable to early NFT deposition (e.g., the [trans‐]entorhinal cortex) exhibit atrophy as early as preclinical AD when measured both through absolute markers as well as their longitudinal rates of chan.^16^, ^17^, ^18^, ^19^, ^20^, ^21^, ^22^, ^23^, ^24^, ^25^ With disease progression, NFTs increasingly occur in medial and posterior parietal structures, where atrophy has been described in prodromal and fully manifest AD.^10^, ^26^, ^27^ Although this previous research suggests that NFT progression patterns are reflected in the timeline of MTL and parietal subregional atrophy, the transferability of these findings to real‐world memory clinic settings is unknown, requiring validation through prospective studies conducted in such specialized centers. Notably, patients with subjective cognitive decline (SCD, i.e., individuals subjectively experiencing memory impairment while performing within normal ranges on standard neuropsychological inventories) were often underrepresented in relevant previous studies, despite their potential as early intervention targets given their early presentation to primary and secondary care.

A biomarker's performance may not just vary by disease stage, but also by intended use. Intended uses for biomarkers are manifold and include diagnosis, prognosis, and disease monitoring.^1^, ^8^, ^28^ Thus, developing sensitive atrophy markers for different intended uses and disease stages requires detailed knowledge of each candidate marker's properties beyond its earliest point of abnormality, including rates of change and relationships with neuropsychological inventories along the disease course.^1^ Although MTL and parietal subregional atrophy markers have been linked to cognitive decline in AD (e.g., see Chauveau et al.,^17^ Xie et al.,^29^ and Eskildsen et al.^30^), no study has systematically examined their associations with a broad range of neuropsychological measures across disease stages and intended uses.

### 1.1 The present study

This study aims to thoroughly characterize MTL and parietal subregional atrophy markers in a prospective, memory clinic–based cohort, addressing different intended uses in a disease stage–dependent manner. First, we assess the progression of the investigated atrophy markers across clinical disease stages, using both cross‐sectional and longitudinal recordings to distinguish accumulated and ongoing atrophy. Our sample spans National Institute on Aging–Alzheimer's Association (NIA‐AA) clinical stages 1–4,^1^ including a group of SCD patients (clinical stage 2) to investigate a potential atrophy signature of subjectively experienced cognitive decline in preclinical AD. Second, we examine how the analyzed atrophy markers develop across data‐driven, continuous disease stages. By integrating clinical and demographic data, this approach enables the investigation of AD progression beyond clinical staging, which is especially valuable in early disease stages, when phenotypical changes are subtle and single biomarkers may be less reliable.^31^ Finally, we investigate the dynamic coupling of atrophy markers and scores on various established neuropsychological inventories for different potential uses, including diagnosis, prognosis, and monitoring at different clinical disease stages.

## 2 METHODS

### 2.1 Participants and clinical disease staging

We included data from 363 participants enrolled in the DZNE Longitudinal Cognitive Impairment and Dementia Study (DELCODE).^32^ This sample comprised cognitively unimpaired individuals (CU) who were Aβ negative (CU Aβ–, *n *= 165) as well as Aβ‐positive participants representing clinical AD stages 1–4 according to the NIA‐AA criteria.^1^ Specifically, this included Aβ‐positive CU (CU Aβ+ [stage 1], *n* = 30) and memory clinic referrals with subjective cognitive decline (SCD Aβ+ [stage 2], *n* = 78), mild cognitive impairment (MCI Aβ+ [stage 3], *n* = 51), and mild dementia of the Alzheimer type (DAT Aβ+ [stage 4], *n* = 39).^33^ The full inclusion criteria are detailed in the Supplementary Methods.

Participants underwent baseline and annual follow‐up assessments including MRI and neuropsychological testing. Given our particular interest in MTL subregions, study visits were only considered if these data were available (see Figure S1 in supporting information for an overview of follow‐up availabilities). This resulted in an average of 3.34 (standard deviation [SD] = 0.81) total assessments per subject with an average follow‐up interval of 1.08 (SD = 0.26) years between study visits. DELCODE was registered with the German Clinical Trials Registry (DRKS; DRKS00007966) prior to inclusion of the first participants.

**RESEARCH IN CONTEXT**

1. **Systematic review**: Previous research has highlighted several shortcomings in the current routine use of structural brain magnetic resonance imaging (MRI) for Alzheimer's disease (AD). Especially given recent advances in disease‐modifying treatments, enhancing sensitivity to early disease stages and establishing robust correlations with both current and future cognitive impairment are essential.
2. **Interpretation**: Our findings indicate that specific, anatomically fine‐grained atrophy markers exhibit abnormal reductions already before the manifestation of cognitive impairment. As the disease progresses, different atrophy markers are best suited for distinct purposes, including AD diagnosis, prognosis, and monitoring. These results provide a framework for selecting the most informative MRI readouts based on the targeted disease stage and intended use.
3. **Future directions**: Future research should validate these findings in independent cohorts and explore the optimal combination of structural MRI markers with other biomarkers for specific applied scenarios.

### 2.2 Fluid biomarkers

Data on Aβ‐positivity were preferentially obtained from lumbar cerebrospinal fluid (CSF; available in *n* = 224, 61.71%, see Supplementary Methods for details on CSF sampling). A threshold for Aβ‐positivity of ≤ 0.08 was calculated through two‐component Gaussian mixture modelling of Aβ42/Aβ40 ratios (Mesoscale Diagnostics LLC). If CSF was unavailable, we calculated individual probabilities of Aβ‐positivity, ranging from 0 to 1 (least to most likely to be Aβ‐positive in CSF). These probabilities were obtained using a binomial logistic regression model, predicting Aβ‐positivity in CSF with age, apolipoprotein E genotype, plasma phosphorylated tau 181 concentration (Simoa assays; Quanterix), and plasma Aβ42/Aβ40 ratios (Lumipulse G System assays; Fujirebio Inc.^34^) included as predictors.^35^ A positivity cutoff of > 0.639 was determined using the Youden index, applying a cost ratio of 1.5:1 for false negatives versus false positives. This decision was guided by evidence suggesting Aβ42/Aβ40 ratios to become abnormal earlier in CSF than in the peripheral blood, making specificity a higher priority than sensitivity.^36^ The model is further described in the Supplementary Methods.

### 2.3 Neuropsychological testing

We included a broad range of measures from the DELCODE neuropsychological testing battery, targeting different cognitive domains. This included tests of episodic memory (Free and Cued Selective Reminding Test–Free + Total Recall [FCSRT‐96^37^], Wechsler Memory Scale IV [WMS‐IV^38^] Logical Memory Delayed Recall, Alzheimer's Disease Assessment Scale‐Cognitive subscale [ADAS‐Cog^39^] delayed and immediate word recall, Face Name Associative Recognition Task), language (sum of animals and groceries verbal fluency, FCSRT Naming), executive functions (Symbol‐Digit Modalities Test [SDMT ^40^], time difference of the Trail Making Test Parts B and A [TMT B–A], a Flanker task^41^, ADAS‐Cog Number Cancellation), working memory (WMS‐R Digit Span), and visuospatial functions (clock drawing and copying, ADAS‐Cog Figure Savings and Copying). In addition, we assessed measures of clinical functioning (Clinical Dementia Rating Sum of Boxes scale [CDR‐SB^42^], Functional Activities Questionnaire [FAQ^43^]), neuropsychiatric symptoms (Neuropsychiatric Inventory–Questionnaire [NPI‐Q^44^]), and cognitive composite scores (ADAS‐Cog13, Mini‐Mental State Examination [MMSE^45^], Preclinical Alzheimer Cognitive Composite [PACC‐5^46^]). If visual inspection of the data indicated that test score distributions strongly deviated from normality, we applied pre‐normalization using latent process modeling implemented in the “lcmm” R package.^47^ Cognitive test scores were *z*‐standardized to participants without manifest cognitive impairment, that is, the CU and SCD groups, and, if needed, inverted so that lower scores represented worse performance across inventories.

### 2.4 MRI acquisition

MRI data were collected on 3 Tesla Siemens MRI system, including a T1‐weighted, 3D whole‐brain magnetization prepared rapid gradient echo sequence (MPRAGE; echo time/repetition time [TE/TR] = 437/2500 ms, inversion time = 1100 ms, 7° flip angle, 1 mm isotropic resolution) and a T2‐weighted turbo spin‐echo (TSE; TE/TR = 354/3500 ms, 120° flip angle, 0.5 × 0.5 × 1.5 mm resolution) sequence. To optimally visualize MTL subregions, TSE images were oriented orthogonally to the longitudinal axis of the hippocampus and were limited to a slab covering the MTL.^48^

### 2.5 MRI processing

Several research questions addressed by this study require the longitudinal assessment of subregional atrophy markers. When performing longitudinal segmentations, processing images individually may introduce unrelated noise. Hence, the current study uses pipelines that minimize and homogenize noise across measurements by using within‐subject templates.^49^

MTL subregional volumes were derived using a newly developed longitudinal implementation of the automated segmentation of hippocampal subfields (ASHS^13^, ^50^) algorithm, using subject‐specific templates. This pipeline can be applied to both T1‐ (T1‐ASHS) and T2‐weighted images (T2‐ASHS). This flexibility is crucial because different structures are best segmented on different image modalities; for example, hippocampal subfields on T2‐weighted and the amygdala on T1‐weighted images.^51^, ^52^ Technical details of this pipeline are summarized in the Supplementary Methods and were described in a previous publication from our group.^2^ T2‐ASHS was used with the Penn ABC 3T atlas, delineating hippocampal subfields (cornu ammonis [CA] 1–3, dentate gyrus, subiculum) as well as the extrahippocampal MTL cortices (entorhinal cortex, Brodmann area [BA] 35 [approximately corresponding to Braak's transentorhinal cortex^50^], BA36, and parahippocampal cortex).^20^, ^53^ To reduce the number of ROIs, we combined the CA2, CA3, and dentate gyrus labels into a single CA23DG label, as these regions are less affected by early AD‐related NFTs.^54^ Hippocampal subfields were only segmented in the anterior hippocampus (combining hippocampal head and body), while its posterior‐most portion was segmented as hippocampal tail. T1‐ASHS with an updated version of the PMC‐T1 atlas was used to generate segmentations of the amygdala.^19^, ^51^ We also conducted standard cross‐sectional T2‐ASHS segmentation of baseline images to evaluate the comparability of longitudinally derived volumes with those generated using the established T2‐ASHS algorithm.

Average cortical thicknesses of the granular retrosplenial cortex (isthmus of the cingulate gyrus), inferior parietal cortex, posterior cingulate cortex, and the precuneus were generated from T1‐weighted images using FreeSurfer's longitudinal stream (v7.1.1, Desikan–Killiany atlas, http://surfer.nmr.mgh.harvard.edu/).^49^

Gray matter volumes were adjusted for their relationship with total intracranial volume in all CU Aβ‐participants included in DELCODE.^55^ All structural measures were *z*‐standardized to the CU Aβ– group. Pipeline availabilities are indicated in Figure S2 in supporting information.

### 2.6 Data‐driven disease staging

A data‐driven disease progression model was trained to capture disease progression on a continuous scale, closely following a previous analysis of DELCODE data.^56^, ^57^ Briefly, the model was trained on all available longitudinal CSF Aβ42/Aβ40 ratios and phosphorylated tau 181 concentrations, as well as PACC‐5 and ADAS‐Cog13 sum scores. While the previously reported model included entorhinal and hippocampal volume, we retrained an otherwise analogous model in the same sample, this time excluding atrophy markers to avoid circularity in our analyses. Using this model, we estimated a continuous disease stage, expressed in years, for each participant's baseline study visit in reference to the entire DELCODE sample. To simplify interpretation of the arbitrarily scaled disease stages, we adjusted the original values for age, sex, and years of education and applied linear transformation to introduce a clinical reference point by setting the median of the CU Aβ+ group to zero.

### 2.7 Statistical analyses

Statistical analyses were performed in R v4.2.2.^58^ The threshold for statistical significance was *P* ≤ 0.05. Two‐way intraclass correlations tested agreement between baseline volumes from longitudinal and cross‐sectional T2‐ASHS. We used analysis of covariance (ANCOVA) to test for effects of study site on scaled atrophy markers within each clinical disease stage. ANCOVAs were also used to compare baseline atrophy markers across clinical disease stages. Post hoc comparisons comprised contrasts of all clinical stages against the CU Aβ– group and contrasts of consecutive clinical stages (e.g., MCI Aβ+ vs. SCD Aβ+). We investigated whether longitudinal trajectories of atrophy markers differed by clinical stage through linear mixed effects models with participant‐level random slopes and intercepts, applying the aforementioned contrasts.

Next, analysis of variance was used to test for differences in data‐driven disease stage between consecutive clinical disease stages at baseline. To model atrophy trajectories along data‐driven disease stages, we estimated longitudinal stages by adding years since baseline to each individual's estimated baseline stage. These projected stages were used in linear mixed‐effects models to predict each atrophy marker. Cubic B‐splines (*df* = 3) were used to capture non‐linear mean trajectories, and the first derivatives of the resulting curves were examined to assess slope changes with disease progression.

Finally, we analyzed the longitudinal relationships of regional structure and cognitive measures using bivariate latent growth curve models (LGCMs), implemented in the “OpenMx” R package.^59^, ^60^ Bivariate LGCMs allow for the estimation of change in two longitudinally recorded variables and the relationships thereof. Models were specified and fitted for each pairing of structural and cognitive variables as visualized in Figure S3 in supporting information. An acceptable model fit was defined by a comparative fit index (CFI) > 0.90 and a root mean square error of approximation (RMSEA) < 0.08. Wald tests (critical *Z* = 1.96) were used to ensure sufficient variance in latent intercepts and slopes to test for covariance. Likelihood ratio tests were used to freely estimate covariance parameters of interest, each reflecting different intended uses for structural MRI markers. This included the baseline–baseline (diagnosis and case finding), baseline–slope (prognosis), and slope–slope (disease monitoring) pairwise associations of structural and cognitive variables. We also investigated if these relationships differed among clinical stages (CU Aβ–, preclinical AD comprising CU Aβ+ and SCD Aβ+, and symptomatic AD comprising MCI Aβ+ and DAT Aβ+). To this end, multigroup models were estimated in which the parameter of interest was freely estimated within each group, constraining all other parameters to be equal across groups. Group‐wise parameters were tested against 0 within each group using likelihood ratio tests.

All analyses controlled for baseline age and sex. Years of education was included as a covariate when predicting cognitive scores. Models including disease time were not corrected for demographics as they were regressed out of this metric.

## 3 RESULTS

An overview of participant characteristics is provided in Table 1. Intra‐class correlation coefficients of baseline volumes generated using the standard cross‐sectional and newly developed longitudinal T2‐ASHS pipelines were excellent, supporting their comparability (*n *= 272, mean intra‐class correlation = 0.94, SD = 0.02; range = 0.90–0.97, all *P*_FDR_ < 0.001; *P* values were corrected across all eight ROIs; Figure S4 in supporting information). There were no statistically significant effects of site on atrophy markers (Table S1, Figure S5 in supporting information).

**TABLE 1:** Characteristics of the analyzed sample.

Diagnostic group							
	Missing	CU Aβ–	CU Aβ+	SCD Aβ+	MCI Aβ+	DAT Aβ+	Total
	*n* (%)	*n *= 165	*n *= 30	*n *= 78	*n *= 51	*n *= 39	*N *= 363
NIA‐AA clinical disease stage		n.a.	1	2	3	4	
Baseline age, years	0 (0)	68.14 (5.05)	71.23 (6.51)	73.07 (5.55)	73.75 (4.82)	74.74 (6.58)	70.95 (6.04)
Sex, female	n.a.	106 (64.24%)	12 (40.00%)	29 (37.18%)	27 (52.94%)	19 (48.72%)	193 (53.17%)
Education level, years	n.a.	14.55 (2.78)	14.27 (3.14)	14.50 (2.98)	14.08 (3.32)	12.97 (2.86)	14.28 (2.96)
Baseline data‐driven disease stage, years	33 (9.09%)	−3.61 (3.26)	−0.92 (3.35)	−0.27 (2.63)	2.32 (2.16)	5.34 (2.31)	−0.93 (4.17)
Number of assessments	n.a.	3.56 (0.70)	3.67 (0.66)	3.17 (0.83)	3.18 (0.87)	2.72 (0.76)	3.34 (0.81)
Source of Aβ status, probabilistic model^a^	n	79 (47.88%)	8 (26.67%)	33 (42.31%)	11 (21.57%)	8 (20.51%)	139 (38.29%)
Pipeline availability							
T1‐ASHS	n.a.	148 (89.70%)	27 (90.00%)	76 (97.44%)	50 (98.04%)	37 (94.87%)	338 (93.11%)
T2‐ASHS	n.a.	151 (91.52%)	28 (93.33%)	61 (78.21%)	32 (62.75%)	18 (46.15%)	290 (79.89%)
FreeSurfer	n.a.	165 (100.00%)	30 (100.00%)	78 (100.00%)	51 (100.00%)	39 (100.00%)	363 (100.00%)
Cognitive test scores^b^							
PACC‐5	37 (10.19%)	0.23 (0.54)	−0.16 (0.74)	−0.36 (0.71)	−1.28 (0.72)	−3.52 (1.35)	−0.30 (1.11)
MMSE	1 (0.28%)	29.57 (0.71)	29.20 (1.06)	29.17 (1.04)	27.84 (1.60)	23.51 (3.36)	28.56 (2.34)
ADAS‐Cog13	9 (2.48%)	5.34 (3.01)	6.92 (4.22)	8.19 (3.93)	16.50 (6.63)	30.22 (7.66)	10.08 (8.86)
CDR‐SB	7 (1.93%)	0.05 (0.21)	0.26 (0.85)	0.51 (0.76)	1.36 (0.96)	4.29 (1.95)	0.80 (1.54)

Abbreviations: Aβ, amyloid beta; ADAS‐Cog13, Alzheimer's Disease Assessment Scale‐Cognitive subscale sum score; ASHS, automated segmentation of hippocampal subfields; CDR‐SB, Clinical Dementia Rating Sum of Boxes; CSF, cerebrospinal fluid; CU, cognitively unimpaired; DAT, dementia of the Alzheimer's disease type; MCI, mild cognitive impairment; MMSE, Mini‐Mental State Examination; n.a., not applicable; NIA‐AA, National Institute on Aging–Alzheimer's Association; PACC‐5, Preclinical Alzheimer Cognitive Composite; SCD, subjective cognitive decline.

^a^ Corresponds to the number of participants with missing CSF data. Aβstatus was directly determined from CSF Aβ42/Aβ40 ratios where available.

^b^ Raw scores are displayed.

### 3.1 Baseline assessments reveal atrophy of early Braak regions in preclinical AD with SCD

**FIGURE 1 alz70482-fig-0001:**
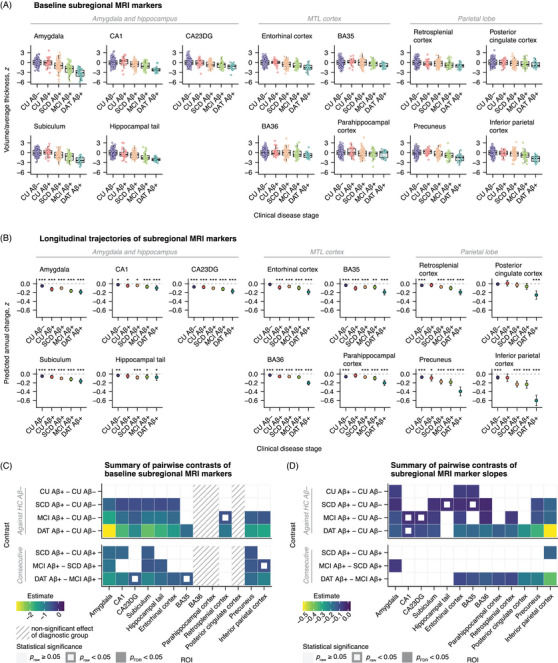
Baseline and longitudinal MTL and parietal subregional atrophy markers across the clinical AD continuum. A, Boxplot diagrams of baseline gray matter volumes and average cortical thicknesses across ROIs and clinical disease stages, grouped by meta‐region. B, Annual change of of atrophy markers across ROIs and clinical disease stages, grouped by meta‐region. The shown slopes were estimated from linear mixed effects models. Error bars denote 95% confidence intervals. Asterisks highlight significant slopes, that is, significant change over time. C, Matrix displaying estimates from analysis of covariance post hoc group comparisons of baseline atrophy markers. The striped pattern indicates markers with non‐significant main effects of clinical disease stage (not passed to post hoc tests). D, Estimates from pairwise stage comparisons of longitudinal atrophy marker slopes. *P < 0.05. **P < 0.01. ***P < 0.001. Aβ, amyloid beta; BA, Brodmann area; CA, cornu ammonis; CA23DG, cornu ammonis 2, 3, and dentate gyrus; CU, cognitively unimpaired; DAT, dementia of the Alzheimer's disease type; FDR, false discovery rate; IPC, inferior parietal cortex; MCI, mild cognitive impairment; MRI, magnetic resonance imaging; MTL, medial temporal lobe; ROI, region of interest; SCD, subjective cognitive decline; SUB, subiculum.

The first main analysis aimed to identify potential baseline differences of atrophy markers among clinical disease stages using ANCOVAs (Figure 1A,C, see also Tables S2 and S3 in supporting information for details on ANCOVA results and post hoc comparisons, respectively). We applied false discovery rate (FDR) correction to *P* values for the effect of clinical stage across atrophy markers (13 comparisons in total), as well as within post hoc comparisons for each atrophy marker (seven comparisons per marker). Atrophy markers from Aβ‐positive groups were first compared to those of CU Aβ– participants and there were no significant differences for the CU Aβ+ group. Meanwhile, volumes of the amygdala (*b* = −0.70, *t* = −3.75, *P*_FDR_ < .001), the entorhinal cortex (*b* = −0.44, *t* = −2.75, *P*_FDR_ = 0.015), and all hippocampal ROIs (all *b* ≤ −0.36, *t* ≤ −2.14, *P*_FDR_ ≤ 0.047) were significantly reduced in the SCD Aβ+ group. The same regional volumes were reduced in MCI Aβ+ participants, though these effects were numerically stronger than in the SCD Aβ+ group (all *b* ≤ −0.59, *t* ≤ −2.97, *P*_FDR_ ≤ 0.011). In addition, MCI Aβ+ participants exhibited significantly reduced cortical thickness in the precuneus (*b* = −0.62, *t* = −2.53, *P*_FDR_ = 0.028) and in the inferior parietal cortex (*b* = −0.51, *t* = −2.45, *P*_FDR_ = 0.035). All structural markers included in the post hoc analysis were significantly reduced in DAT Aβ+ patients (all *b* ≤ −0.80, *t* ≤ −3.18, *P*_FDR_ ≤ 0.011). Analyzing further contrasts representing consecutive clinical stages, we observed significant differences across comparisons (SCD Aβ+ vs. CU Aβ+, MCI Aβ+ vs. SCD Aβ+, DAT Aβ+ vs. MCI Aβ+). These were mainly focused on the amygdala, hippocampal ROIs, the precuneus, and the inferior parietal cortex.

Clinical disease stage had a non‐significant effect on BA36 and parahippocampal cortex volume, as well as on posterior cingulate cortical thickness. These models were not passed to post hoc analysis.

### 3.2 Longitudinal MRI reveals accelerated atrophy in preclinical AD

Incorporating data from follow‐up MRI assessments, we tested if clinical disease stages differed in their associated longitudinal trajectories of atrophy markers using linear mixed effect models. Random slopes showed insufficient variance when predicting posterior cingulate cortical thickness and were therefore omitted from this specific model. Estimated slopes (i.e., annual rates of change of atrophy markers) are displayed by clinical disease stage in Figure 1B and Figure 1D summarizes the results of their pairwise comparisons (see also Tables S4–6 in supporting information; FDR correction was performed among all seven planned contrasts per atrophy marker). In CU Aβ– participants, a significant decline was observed across markers except for entorhinal volume and posterior cingulate cortical thickness. Decline was further amplified in several regions within the different Aβ‐positive groups. While no significant effects were observed for the CU Aβ+ group in the cross‐sectional analysis, there was an accelerated volumetric decline over time in the amygdala (*b* = −0.07, *t* = −2.99, *η* ^2^_partial_ = 0.03, *P*_FDR_ = 0.007), entorhinal cortex (*b* = −0.07, *t* = −3.01, *η* ^2^_partial_ = 0.04, *P*_FDR_ = 0.007), and BA35 (*b* = −0.07, *t* = −2.73, *η* ^2^_partial_ = 0.03, *P*_FDR_ = 0.016). The SCD Aβ+ group exhibited an even more pronounced decline relative to the CU Aβ– group, particularly in parietal subregions (precuneus: *b* = −0.10, *t* = −3.10, *η* ^2^_partial_ = 0.04, *P*_FDR_ = 0.005; inferior parietal cortex: *b* = −0.15, *t* = −3.89, *η* ^2^_partial_ = 0.06, *P*_FDR_ < 0.001). Additional regions within the medial temporal lobe (amygdala, subiculum, entorhinal cortex, and BA36) also showed significantly faster decline in the SCD Aβ+ group. Comparing SCD Aβ+ and CU Aβ+ participants, the only significant difference was in the rate of thinning in the inferior parietal cortex (*b* = −0.15, *t* = −2.74, *η* ^2^_partial_ = 0.03, *P*_FDR_ = 0.009). A similar pattern to that observed in SCD Aβ+ individuals was found in the MCI Aβ+ group, with an additional acceleration of thinning in the retrosplenial cortex (*b* = −0.06, *t* = −2.67, *η* ^2^_partial_ = 0.03, *P*_FDR_ = 0.027). In DAT Aβ+ participants, nearly all structures, except for the hippocampal tail and CA1, showed accelerated atrophy. Compared to MCI Aβ+ participants, structural decline in DAT Aβ+ individuals progressed more rapidly in extrahippocampal and parietal regions, while hippocampal subfields and the amygdala showed no significant differences.

### 3.3 Data‐driven disease staging offers detailed insights into subregional atrophy progression

**FIGURE 2:**
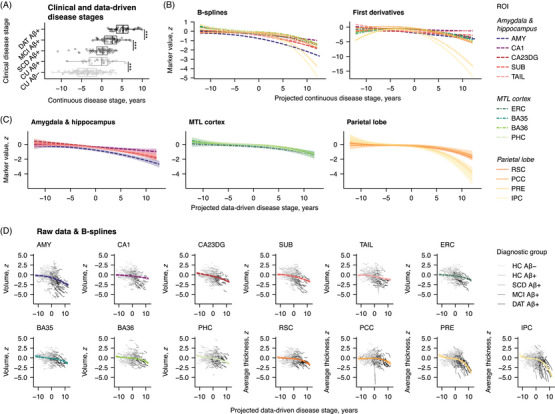
Non‐linear changes in MTL and parietal subregional atrophy markers along continuous, data‐driven disease stages. A, Boxplot diagrams of baseline data‐driven disease stages by clinical disease stage, centered to the median of the CU Aβ+ group. B, Cubic B‐splines and their first derivatives were used to assess the development of atrophy markers across data‐driven disease stages. The estimated trajectories with bootstrapped confidence intervals are also shown in (C), where they are clustered by meta‐region. D, For each atrophy marker, the predicted curves are plotted along with the observed participant‐level trajectories. ***P < 0.001. Aβ, amyloid beta; AMY, amygdala; BA; Brodmann area; CA, cornu ammonis; CA23DG, cornu ammonis 2, 3, and dentate gyrus; CU, cognitively unimpaired; DAT, dementia of the Alzheimer's disease type; ERC, entorhinal cortex; HC, healthy control; IPC, inferior parietal cortex; MCI, mild cognitive impairment; MTL, medial temporal lobe; PCC, posterior cingulate cortex; PHC, parahippocampal cortex; PRE, precuneus; ROI, region of interest; RSC, retrosplenial cortex; SCD, subjective cognitive decline; SUB, subiculum; TAIL, hippocampal tail.

Independent of discrete clinical staging, we next analyzed the evolution of regional atrophy markers in relation to continuous, data‐driven disease stages. The disease progression model used to derive individual stages is illustrated in Figure S6 in supporting information. We found that baseline stage increased with clinical progression (i.e., with each successive clinical disease stage), except that there was no significant difference between CU Aβ+ and SCD Aβ+ participants (*b* = 0.65, *P*_FDR_ = 0.308; otherwise range [*b*]: 2.58–3.02, all *P*_FDR_ < 0.001;[Fig alz70482-fig-0001] Figure 2A, four FDR‐corrected comparisons). Linear mixed effects models with B‐splines were used to capture non‐linearity in the longitudinal trajectories of atrophy markers with data‐driven disease stage. Figure 2B–D show the fitted slopes and their first derivatives. As expected in a sample of older adults, progressive atrophy (i.e., negative first derivatives) was evident already at early stages below the CU Aβ+ group median, which was set to be zero. Within the MTL, the amygdala exhibited the most pronounced decline across data‐driven disease stages. Interestingly, the first derivatives of the fitted curves indicate that atrophy rates of hippocampal subfields and the amygdala stabilized toward later stages. Atrophy rates in the MTL cortex and parietal lobe were low in early stages but accelerated in stages primarily observed in symptomatic individuals, with particularly sharp increases in the precuneus and inferior parietal cortex.

### 3.4 Structural and cognitive changes covary uniquely at different clinical disease stages

**FIGURE 3:**
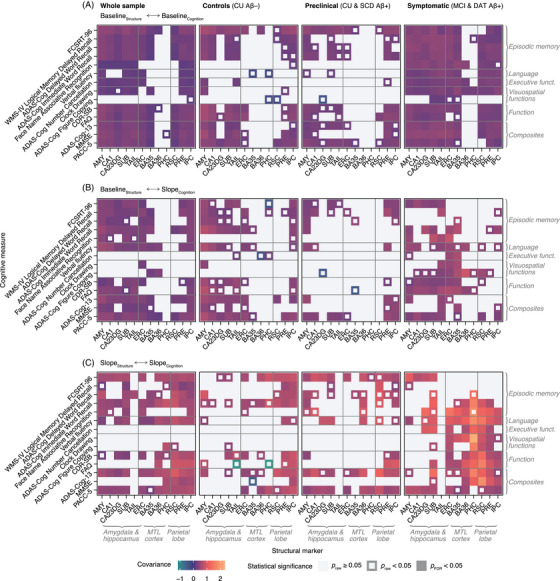
Estimates from bivariate LGCMs capturing the disease stage–specific cross‐sectional and longitudinal relationships of subregional atrophy markers and neuropsychological test scores. The covariance parameters of interest represented the (A) baseline–baseline, (B) baseline–slope, and (C) slope–slope pairwise associations of atrophy markers and neuropsychological test scores. Aβ, amyloid beta; ADAS‐Cog, Alzheimer's Disease Assessment Scale‐Cognitive subscale; AMY, amygdala; BA, Brodmann area; CA, cornu ammonis; CA23DG, cornu ammonis 2, 3, and dentate gyrus; CSD‐SB, Clinical Dementia Rating Sum of Boxes scale; ERC, entorhinal cortex; FAQ, Functional Activities Questionnaire; FCSRT, Free and Cued Selective Reminding Test; FDR, false discovery rate; IPC, inferior parietal cortex; LGCM, latent growth curve model; MMSE, Mini‐Mental State Examination; MTL, medial temporal lobe; PACC‐5, Preclinical Alzheimer Cognitive Composite; PHC, parahippocampal cortex; PRE, precuneus; RSC, retrosplenial cortex; SUB, subiculum; TAIL, hippocampal tail; WMS‐IV, Wechsler Memory Scale IV.

Finally, we analyzed longitudinal measures of regional brain structure and cognition to identify their potentially disease stage–specific couplings. To this end, bivariate LGCMs were fitted both in the whole sample and in subsamples representing different clinical disease stages. Due to their non‐normal distributions, ADAS‐Cog Delayed Word Recall, ADAS‐Cog Figure Copying, ADAS‐Cog13, CDR‐SB, Clock Copying, Clock Drawing, FAQ, MMSE, and NPI‐Q scores were pre‐normalized using latent process modelling (Supplementary Results, Figures S7 and S8 in supporting information). Most models showed adequate fit and variance structures. However, FCSRT Naming SDMT, TMT B–A, Flanker, WMS‐R Digit Span, Clock Copying, and NPI‐Q scores, as well as posterior cingulate cortical thicknesses, had insufficient slope variances and are therefore not reported. Additional details on model fit are reported in the Supplementary Results and Figures S9 and S10 in supporting information. The estimates for all covariance parameters of interest are visualized in Figure 3. We applied FDR correction across all statistical tests performed in each analyzed group (3 parameters of interest ⨉ 14 test scores ⨉ 12 atrophy markers = 504 comparisons per group).

As expected, all significant covariance estimates were positive, indicating that structural decline was linked to worsening cognition. However, no consistent covariance pattern emerged across clinical disease stages, with each group showing distinct associations between subregional atrophy markers and individual test scores.

Clinical disease stage‐specific covariance matrices revealed that significant estimates were more frequent and descriptively higher in the symptomatic group (MCI Aβ+ and DAT Aβ+) compared to the CU Aβ– and preclinical (CU Aβ+ and SCD Aβ+) groups, reflecting stronger structure‐cognition coupling at later clinical stages. Structure‐cognition relationships were numerically weaker in cross‐sectional analyses (baseline‐baseline, Figure 3A) than in analyses where structure was assessed cross‐sectionally and cognition longitudinally (baseline‐slope, Figure 3B). Covariances were numerically largest when both structure and cognition were assessed longitudinally (slope‐slope, Figure 3C), suggesting that brain structure and cognition are more closely linked through their changes over time than through their baseline measures.

A notable shift was observed in regional patterns comparing baseline–slope and slope–slope associations: When brain structure is considered longitudinally instead of cross‐sectionally, significant covariances are increasingly focused on the structures in the MTL cortices and the parietal lobe. This pattern occurred across groups but was most pronounced in symptomatic participants.

In preclinical stages, the FCSRT‐96 and PACC‐5 demonstrated the most consistent associations across covariance parameters and regions, with the FCSRT‐96 showing more widespread and significant structural correlates than other episodic memory measures. Significant slope–slope associations were also observed in models including the ADAS‐Cog13. Functional decline was linked to thinning in the precuneus and inferior parietal cortex, while sign associations with language, executive functioning, or visuospatial scores were rare.

In symptomatic groups, cross‐sectional associations between structural and cognitive measures were notably broad, with only parahippocampal cortex and BA36 volumes showing limited associations. Baseline–slope relationships were primarily centered on MTL structures across cognitive domains, while slope–slope covariances focused on the MTL cortex and parietal lobe, encompassing cognitive domains beyond episodic memory and cognitive composites.

## 4 DISCUSSION

This study provides a detailed characterization of MTL and parietal subregional atrophy along the AD continuum, revealing abnormally accelerated atrophy in MTL subregions already during preclinical disease stages. Notably, the presence of SCD in preclinical AD was associated with a widespread pattern of manifest MTL atrophy. Independent of clinical staging, the examined atrophy markers uniquely developed within a data‐driven disease staging framework, with amygdalar volume showing the earliest decline. Finally, we found that associations between atrophy markers and neuropsychological test scores varied by clinical disease stage and intended use.

Overall, the atrophy patterns identified using both clinical and data‐driven AD staging mirrored the Braak staging framework of NFT progression, with the MTL being affected prior to the parietal cortex.^10^, ^14^ Our results thus align with previous findings on the co‐localization of NFTs and atrophy, as well as with studies on MRI signatures of early AD, further supporting the use of structural MRI as a biomarker for AD‐related neurodegeneration.^3^, ^9^, ^10^, ^11^, ^24^, ^25^, ^61^ The additional insights generated by our study as well as its limitations are discussed in the following.

### 4.1 Accelerated atrophy is detectable in preclinical AD

Our study underscores the value of subregional atrophy assessments, particularly when targeting early disease stages. For instance, our longitudinal data suggest that in preclinical AD, atrophy spares the posterior MTL (parahippocampal cortex, hippocampal tail), while anterior structures deteriorate earlier. Another example, now at the level of anatomical substructures, is given by our longitudinal analyses across clinical disease stages: here, abnormal atrophy first emerged in the anterior MTL cortices (entorhinal cortex, BA35, BA36) and amygdala at clinical stage 1 (CU Aβ+), before hippocampal subfields showed accelerated atrophy at clinical stage 2 (SCD Aβ+). While the MTL cortices are known as early hotspots of AD‐related NFTs and atrophy, the amygdala has been underrepresented in relevant research.^62^ Alongside our findings, only few studies suggest that amygdalar volume may be more sensitive to early AD than the commonly used hippocampal volume.^63^, ^64^ Interestingly, hippocampal and amygdalar atrophy rates stabilized toward later data‐driven disease stages and did not differ between DAT Aβ+ and MCI Aβ+ participants, indicating reduced sensitivity in manifest AD.

Crucially, our study reinforces the relevance of distinguishing clinical stages 1 (CU Aβ+) and 2 (SCD Aβ+) as manifest MTL atrophy as well as accelerated atrophy rates of the inferior parietal cortex and the precuneus were only observed in the SCD Aβ+ but not the CU Aβ+ group. These findings align with studies linking SCD in preclinical AD to elevated biomarkers of AD proteinopathy and an elevated risk of progression to cognitive impairment.^32^, ^65^, ^66^, ^67^, ^68^

### 4.2 Cross‐sectional and longitudinal atrophy markers provide distinct insights into disease progression

Our findings show that cross‐sectional and longitudinal atrophy markers provide distinct, yet complementary, insights into accumulated and ongoing regional atrophy, respectively. For example, in SCD Aβ+ participants, precuneus and inferior parietal cortical thickness appeared normal cross‐sectionally but showed accelerated atrophy over time in longitudinal assessments. Meanwhile, significant accumulated atrophy of these regions was evident in the subsequent MCI Aβ+ stage. While most findings align with this pattern of accelerated decline (ongoing atrophy) preceding cross‐sectional reductions (accumulated atrophy), some results deviate from this trend. For example, our cross‐sectional analyses identified reduced volumes in hippocampal subfield CA1 at the SCD Aβ+ stage while no clinical disease stage was linked with longitudinally accelerated CA1 atrophy rates. Importantly, we believe it is not paradoxical that atrophy rates in BA35, BA36, and the parahippocampal cortex accelerate during preclinical stages despite the absence of significant cross‐sectional abnormalities before the DAT Aβ+ stage. This can be attributed to the high inter‐individual macro‐anatomical differences in these structures, adding variance to absolute volumetric measures unrelated to neurodegeneration.^50^ Longitudinal within‐subject volumetric analyses account for this variance, making them better suited to capture subtle atrophy in these structures.^49^

### 4.3 Brain structure and cognition show disease stage–specific couplings

Covariance patterns between MRI markers and neuropsychological test scores consistently linked atrophy to cognitive decline, though associations varied by disease stage and covariance parameter.

Building on prior reports of varying sensitivities of cognitive inventories with AD progression, we found that relationships of subregional atrophy markers and neuropsychological test scores varied by clinical disease stage.^69^, ^70^ Overall, we observed stronger and more significant atrophy–cognition associations in symptomatic compared to preclinical AD participants. This finding likely reflects greater intra‐ and interindividual variability in atrophy and cognitive performance in symptomatic AD. It also suggests that early cognitive decline may be more driven by factors beyond atrophy, such as functional alterations or synaptic dysfunction.

The analyzed covariance parameters of atrophy markers and neuropsychological test scores represent three distinct uses for biomarkers: baseline–baseline covariances are relevant for diagnostic and case‐finding purposes, baseline–slope covariances reflect the prognostic value of an atrophy marker, and slope–slope covariances indicate an atrophy marker's suitability for monitoring disease progression. Generally, we observed the strongest covariances among slope–slope associations, suggesting that regional brain structure and neuropsychological test scores are most closely linked in their longitudinal changes, aligning with previous studies.^71^, ^72^, ^73^ The complex interplay of disease stage and intended use can be illustrated by the associations of CA1 volume and FCSRT‐96 scores. The two variables covaried at baseline in both preclinical and symptomatic AD, with stronger covariance in the latter. Regarding prognosis, lower baseline CA1 volume predicted FCSRT‐96 decline only in symptomatic AD. Meanwhile, longitudinal changes in both variables covaried significantly in both groups, with stronger coupling in preclinical AD. These results suggest that CA1 atrophy related to FCSRT‐96 performance emerges mainly during preclinical AD. In symptomatic AD, accumulated CA1 atrophy predicts further decline. However, this later decline appears to be primarily coupled with ongoing atrophy in other MTL subregions, including the amygdala and subiculum.

Our results capture established anatomo–clinical relationships and highlight their dynamic fluctuations with disease progression. For example, cognitive decline was linked to emerging parietal atrophy more strongly in symptomatic than in preclinical AD, aligning with reports of increasing parietal NFT pathology as the disease progresses.^10^, ^74^ In addition, the significant slope–slope relationships of FCSRT‐96 and verbal fluency scores with MTL volumes in preclinical AD complement the established role of the MTL in episodic and semantic memory.^61^, ^75^, ^76^ Interestingly, the FCSRT‐96 stood out for its consistent associations with MTL structure, aligning with prior studies linking it to early tau burden in preclinical AD and highlighting its value among PACC‐5 subtests for distinguishing preclinical AD from healthy aging over time.^46^, ^77^ In contrast, many episodic memory measures (e.g., ADAS‐Cog Delayed Word Recall, WMS‐IV Logical Memory Delayed Recall), showed weak associations with MTL structure. Among cognitive composites, the MMSE was largely unrelated to the analyzed atrophy markers in preclinical AD, while significant associations were found for the ADAS‐Cog13 and PACC‐5. These composites were designed to specifically capture AD‐related cognitive decline and emphasize episodic memory, which may explain their relatively early associations with MTL atrophy.

While focusing on AD, our study also provides insights into brain structure–cognition relationships in healthy aging (CU Aβ–). Contrasting earlier studies, we found no widespread longitudinal relationships between MTL structure and cognition, particularly episodic memory.^78^ This may reflect a lag between atrophy and cognitive decline, as indicated by broader associations of baseline MTL structure with future cognitive decline.

### 4.4 Limitations

Our results should be interpreted given the following limitations. First, our study is strongly motivated by an assumed local association between NFT pathology and atrophy. However, due to lacking regional NFT markers, we were unable to directly evaluate this assumption. Varying regional vulnerability, comorbid pathologies (e.g., TDP‐43, α‐synuclein), and distant NFT effects may have contributed to our findings.^9^ Future studies should investigate the pathobiological processes underlying our results, such as through the use of biomarkers and biological disease staging. Second, our analyses of structure–cognition relationships cannot answer questions of possible mediating or moderating mechanisms, including functional alterations and neuroinflammatory responses. Third, disparities in sample sizes across diagnostic groups may have led to underpowered analyses, emphasizing the importance of the reported effect sizes. Finally, future studies should aim to replicate our findings in independent, more diverse cohorts that include data on race and ethnicity, which were not collected here due to German data protection laws.

## 5 CONCLUSION

Our study offers detailed insights into the dynamics of anatomically detailed atrophy markers within the MTL and parietal lobe across the AD continuum. We show that abnormalities on structural MRI do not necessarily appear imminent to cognitive impairment onset. Instead, accelerated volumetric decline in the entorhinal cortex, BA35, and the amygdala was observed already in preclinical AD. However, these markers appear less effective during symptomatic stages, when disease progression leads to more pronounced atrophy in hippocampal and parietal subregions. We demonstrate that atrophy–cognition associations are disease stage–specific and vary by the intended use of an atrophy marker. Crucially, these associations were highly heterogeneous even within broader brain regions and cognitive domains, such as among MTL subregions and episodic memory tests. These results indicate that achieving optimal use of structural MRI for atrophy assessment—whether in research, clinical practice, or intervention trials—requires markers that are tailored to context‐specific factors, including the intended use and the targeted disease stage.

## CONFLICT OF INTEREST STATEMENT

J.W. has been an honorary speaker for Beeijing Yibai Science and Technology Ltd, Eisai, Gloryren, Janssen, Pfizer, Med Update GmbH, Roche, Lilly, Roche Pharma; has been a member of the advisory boards of Abbott, Biogen, Boehringer Ingelheim, Lilly, Immungenetics, MSD Sharp‐Dohme, Noselab, Roboscreen, and Roche Pharma; and receives fees as a consultant for Immungenetics, Noselab, and Roboscreen. J.W. holds the following patents: PCT/EP 2011 001724 and PCT/EP 2015 052945. All other authors report no conflicts of interest relevant to this work. Author disclosures are available in the supporting information.

## CONSENT STATEMENT

All participants provided their written informed consent to participate in the study in accordance with the Declaration of Helsinki. The local institutional review boards at all participating study sites approved the study protocol.

## Supporting information



Supporting Information

Supporting Information
