# Supplementary material for: Disease stage–specific atrophy markers in Alzheimer's disease
Source: Alzheimers Dement. 2025 Jul 21;21(7):e70482. doi: 10.1002/alz.70482 (PMC12279471; doi:10.1002/alz.70482)
Supplement: Supplementary file 1 — Supporting Information [file ALZ-21-e70482-s001.docx]

Supplementary material

# DELCODE study group

## Group members

| **Name** | **Affiliation** | **Name** | **Affiliation** | **Name** | **Affiliation** |
| --- | --- | --- | --- | --- | --- |
| Holger Amthauer | 21 | Ina Vogt | 4 | Daniela Frimmer | 16 |
| Arda Can Cetindag | 2 | Michael Wagner | 4,6 | Brigitte Huber | 16 |
| Nicoleta Carmen Cosma | 2 | Steffen wolfsgruber | 4 | Daniel Janowitz | 16 |
| Dominik Diesing | 2 | Claudia Bartels | 9 | Max Kreuzer | 16 |
| Marie Ehrlich | 2 | Peter Dechent | 27 | Claudia Müller |  |
| Ersin Ersözlü | 1,2 | Niels Hansen | 9 | Axel Rominger |  |
| Frederike Fenski | 2 | Lina Hassoun | 9 | Jennifer Schmid | 16 |
| Silka Dawn Freiesleben | 2 | Sina Hirschel | 9 | Anna Seegerer | 16 |
| Manuel Fuentes | 1,2 | Sabine Nuhn |  | Julia Stephan |  |
| Dietmar Hauser | 2 | Ilona Pfahlert | 9 | Adelgunde Zollver | 16 |
| Nicole Hujer | 2 | Lena Rausch |  | Lena Burow | 23 |
| Enise Irem Incesoy | 2 | Björn Schott | 8,9,60 | Sylvia de Jonge | 23 |
| Christian Kainz | 22 | Jens Wiltfang | 8,9 | Peter Falkai | 23 |
| Catharina Lange | 2 | Heike Zech | 8,9 | Natalie Garcia Angarita | 23 |
| Katja Lindner | 2 | Abdelmajid Bader | 10 | Thomas Görlitz | 23 |
| Herlind Megges | 1,2 | Juan Carlos Baldermann | 10 | Selim Üstün Gürsel | 23 |
| Oliver Peters | 1,2 | Alexander Drzezga | 35 | Ildiko Horvath | 15 |
| Lukas Preis | 2 | Nasim Roshan Ghiasi | 10 | Carolin Kurz | 23 |
| Slawek Altenstein | 1,3 | Katja Hardenacke | 10 | Eva Meisenzahl-Lechner |  |
| Andrea Lohse |  | Frank Jessen | 4,1 | Robert Perneczky | 15,23,24,25 |
| Christiana Franke | 3 | Hannah Lützerath | 10 | Julia Utecht | 23 |
| Josef Priller | 1,3 | Franziska Maier | 10 | Martin Dyrba | 17 |
| Eike Spruth | 3 | Anja Martikke | 10 | Heike Janecek-Meyer | 18 |
| Miriam Barkhoff | 4 | Dix Meiberth | 10 | Ingo Kilimann | 17 |
| Henning Boecker | 4 | Ayda Rostamzadeh | 10 | Chris Lappe | 18 |
| Frederic Brosseron | 4 | Lena Sannemann | 10 | Esther Lau | 17 |
| Marcel Daamen | 4 | Ann-Katrin Schild | 10 | Henrike Pfaff | 18 |
| Tanja Engels | 4 | Susanne Sorgalla | 10 | Petr Sabik | 17 |
| Jennifer Faber | 4,7 | Simone Stockter | 10 | Monika Schmidt | 17 |
| Klaus Fließbach | 4 | Manuela Thelen | 10 | Heike Schulz | 17 |
| Ingo Frommann | 4 | Maike Tscheuschler | 10 | Sarah Schwarzenboeck |  |
| Marcus Grobe-Einsler | 4,7 | Franziska Uhle | 10 | Stefan Teipel | 17,18 |
| Guido Hennes | 4 | Philip Zeyen | 10 | Marc-Andre Weber |  |
| Gabi Herrmann | 4 | Laura Dobisch | 11 | Martina Buchmann |  |
| Lorraine Jost | 4 | Emrah Düzel | 11 | Tanja Heger | 20 |
| Pascal Kalbhen | 6 | Doreen Grieger-Klose | 11 | Petra Hinderer | 19 |
| Okka Kimmich | 4,7 | Deike Hartmann | 11 | Elke Kuder-Buletta | 19 |
| Xenia Kobeleva | 4 | Coraline Metzger | 11 | Christoph Laske | 19,2 |
| Barbara Kofler |  | Christin Ruß | 11 | Matthias Munk | 19,2 |
| Cornelia McCormick | 4 | Franziska Schulze | 11 | Christian Mychajliw | 19,2 |
| Lisa Miebach | 4 | Oliver Speck | 11 | Sebastian Sodenkamp | 19,50 |
| Carolin Miklitz | 4 | Glanz Wenzel | 11 | Surjo Soekadar |  |
| Demet Oender | 4,7 | Renat Yakupov | 11 | Patricia Sulzer | 20 |
| Sandra Röske | 4 | Gabriel Ziegler | 11 | Theresia Trunk |  |
| Christine Schneider | 4 | Katharina Bürger | 15,16 | Luisa Schneider | 1,2,58,61 |
| Anja Schneider | 4,6 | Lisa Coloma Andrews | 16 | Xiao Wang | 1,2,58,61 |
| Annika Spottke | 4,7 | Martin Dichgans | 15,16 | Kai Shao | 4,62 |
| Melina Stark | 4,6 | Birgit Ertl-Wagner | 26 |  |  |

## Group affiliations

| 1 | German Center for Neurodegenerative Diseases (DZNE), Berlin, Germany |
| --- | --- |
| 2 | Charité – Universitätsmedizin Berlin, corporate member of Freie Universität Berlin and Humboldt-Universität zu Berlin-Institute of Psychiatry and Psychotherapy |
| 3 | Department of Psychiatry and Psychotherapy, Charité, Charitéplatz 1, 10117 Berlin, Germany |
| 4 | German Center for Neurodegenerative Diseases (DZNE), Bonn, Venusberg-Campus 1, 53127 Bonn, Germany |
| 5 | Department of Psychiatry and Psychotherapy, University of Bonn, Venusberg-Campus 1, 53127 Bonn, Germany |
| 6 | Department of Old Age Psychiatry and Cognitive Disorders, University Hospital Bonn and University of Bonn, Bonn, Germany |
| 7 | Department of Neurology, University of Bonn, Venusberg-Campus 1, 53127 Bonn, Germany |
| 8 | German Center for Neurodegenerative Diseases (DZNE), Goettingen, Germany |
| 9 | Department of Psychiatry and Psychotherapy, University Medical Center Goettingen, University of Goettingen, Von-Siebold-Str. 5, 37075 Goettingen |
| 10 | Department of Psychiatry, University of Cologne, Medical Faculty, Kerpener Strasse 62, 50924 Cologne, Germany |
| 11 | German Center for Neurodegenerative Diseases (DZNE), Magdeburg, Germany |
| 12 | Institute of Cognitive Neurology and Dementia Research (IKND), Otto-von-Guericke University, Magdeburg, Germany |
| 13 | Department of Psychiatry and Psychotherapy, Otto-von-Guericke University, Magdeburg, Germany |
| 14 | Department of Neurology, Otto-von-Guericke University, Magdeburg, Germany |
| 15 | German Center for Neurodegenerative Diseases (DZNE, Munich), Feodor-Lynen-Strasse 17, 81377 Munich, Germany |
| 16 | Institute for Stroke and Dementia Research (ISD), University Hospital, LMU Munich, Feodor-Lynen-Strasse 17, 81377 Munich, Germany |
| 17 | German Center for Neurodegenerative Diseases (DZNE), Rostock, Germany |
| 18 | Department of Psychosomatic Medicine, Rostock University Medical Center, Gehlsheimer Str. 20, 18147 Rostock |
| 19 | German Center for Neurodegenerative Diseases (DZNE), Tübingen, Germany |
| 20 | Section for Dementia Research, Hertie Institute for Clinical Brain Research and Department of Psychiatry and Psychotherapy, University of Tübingen, Tübingen, Germany |
| 21 | Charité – Universitätsmedizin Berlin, corporate member of Freie Universität Berlin, Humboldt-Universität zu Berlin, and Berlin Institute of Health, Department of Nuclear Medicine, Augustenburger Platz 1, 13353 Berlin |
| 22 | Center for Cognitive Neuroscience Berlin (CCNB), Department of Education and Psychology, Freie Universität Berlin, Berlin, Germany |
| 23 | Department of Psychiatry and Psychotherapy, University Hospital, LMU Munich, Munich, Germany |
| 24 | Munich Cluster for Systems Neurology (SyNergy) Munich, Munich, Germany |
| 25 | Ageing Epidemiology Research Unit (AGE), School of Public Health, Imperial College London, London, UK |
| 26 | Institute for Clinical Radiology, Ludwig-Maximilians-University, Marchioninistr. 15, 81377 Munich |
| 27 | MR-Research in Neurosciences, Department of Cognitive Neurology, Georg-August-University Goettingen, Germany |
| 28 | Bernstein Center for Computational Neuroscience, Charité — Universitätsmedizin, Berlin, Germany |
| 29 | Department for Biomedical Magnetic Resonance, University of Tübingen, 72076 Tübingen, Germany |
| 30 | Study Center Bonn, Medical Faculty, Venusberg-Campus 1, 53127 Bonn, Germany |
| 31 | Department of Medical Imaging, University of Toronto, Toronto, Canada |
| 32 | Department of Nuclear Medicine, Charité - Universitätsmedizin Berlin, corporate member of Freie Universität Berlin, Humboldt-Universität zu Berlin, and Berlin Institute of Health, Berlin, Germany |
| 33 | Department of Diagnostic and Interventional Radiology and Nuclear Medicine, University Medical Center Hamburg-Eppendorf, Hamburg, Germany |
| 34 | Department of Nuclear Medicine, University Hospital Bonn, Bonn, Germany |
| 35 | Department of Nuclear Medicine, Faculty of Medicine and University Hospital Cologne, University of Cologne, Germany |
| 36 | Department of Nuclear Medicine, Ludwig-Maximilian-University Munich, Munich, Germany |
| 37 | Department of Nuclear Medicine, Rostock University Medical Centre, Rostock |
| 38 | Department of Nuclear Medicine and Clinical Molecular Imaging, Eberhard-Karls-University, Tuebingen, Germany |
| 39 | Excellence Cluster on Cellular Stress Responses in Aging-Associated Diseases (CECAD), University of Cologne, Joseph-Stelzmann-Strasse 26, 50931Köln, Germany |
| 40 | Neurosciences and Signaling Group, Institute of Biomedicine (iBiMED), Department of Medical Sciences, University of Aveiro, Aveiro, Portugal |
| 41 | Division of Neurogenetics and Molecular Psychiatry, Department of Psychiatry and Psychotherapy, Faculty of Medicine and University Hospital Cologne, University of Cologne, Cologne, Germany |
| 42 | Institute for Medical Biometry, Informatics and Epidemiology, University Hospital Bonn, Venusberg-Campus 1, D-53127 Bonn |
| 43 | Department of Neuroradiology, University Hospital Bonn, Bonn, Germany |
| 44 | Clininical Functional Imaging Group, Department of Diagnostic and Interventional Radiology, University Hospital Bonn, Bonn, Germany |
| 45 | School of Medicine, Technical University of Munich; Department of Psychiatry and Psychotherapy, Munich, Germany |
| 46 | University of Edinburgh and UK DRI, Edinburgh, UK |
| 47 | Department of Psychiatry & Glenn Biggs Institute for Alzheimer’s and Neurodegenerative Diseases, San Antonio, TX, USA |
| 48 | Glenn Biggs Institute for Alzheimer's and Neurodegenerative Diseases, UT Health San Antonio, San Antonio, Texas, USA. |
| 49 | Berlin Center for Advanced Neuroimaging, Charité – Universitätsmedizin Berlin, Berlin, Germany |
| 50 | Department of Psychiatry and Psychotherapy, University of Tübingen, Tübingen, Germany |
| 51 | Department of Nuclear Medicine, University Hospital Bonn, Bonn, Germany |
| 52 | Sheffield Institute for Translational Neuroscience (SITraN), University of Sheffield, Sheffield, UK |
| 53 | Department of Neuroradiology, University Hospital LMU, Munich, Germany |
| 54 | Institute of Neuroscience and Medicine (INM-2), Molecular Organization of the Brain, Forschungszentrum Jülich, Germany |
| 55 | Department of Nuclear Medicine, Inselspital, Bern University Hospital, University of Bern, Switzerland |
| 56 | Department for Psychiatry and Psychotherapy, University Clinic Magdeburg, Magdeburg, Germany |
| 57 | Luxembourg Centre for Systems Biomedicine (LCSB), University of Luxembourg, L-4367 Belvaux |
| 58 | Charité Universitätsmedizin Berlin, Department of Psychiatry and Neurosciences, Campus Benjamin Franklin |
| 59 | German Center for Mental Health (DZPG), partner site Berlin |
| 60 | Leibniz Institute for Neurobiology, Brenneckestr. 6, 39118, Magdeburg, Germany |
| 61 | Charité – Universitätsmedizin Berlin, ECRC Experimental and Clinical Research Center, Berlin, Germany |
| 62 | Department of Neurology, XuanWu Hospital of Capital Medical University, Changchun Street 45, 100053, Beijing, China |
| 63 | Division of Translational Genomics of Neurodegenerative Diseases, Hertie Institute for Clinical Brain Research and Center of Neurology, University of Tübingen, Tübingen, Germany |
| 64 | Department of Psychiatry and Psychotherapy, School of Medicine and Health, Technical University of Munich, and German Center for Mental Health (DZPG), Munich, Germany |

# Supplementary methods

## DELCODE inclusion criteria

DELCODE comprises two groups of cognitively unimpaired (CU) participants and three patient groups. Clinical screening of patient groups (subjective cognitive decline [SCD], mild cognitive impairment [MCI], and dementia of the Alzheimer type [DAT]) was performed at the respective memory clinic. Those in the SCD group fulfilled research criteria for SCD, expressed concerns about a subjectively perceived cognitive decline to a physician at the respective memory clinic, and were cognitively unimpaired as defined by a score better than −1.5 *SD* below the German age, sex, and education-adjusted norms for the Consortium to Establish a Registry for Alzheimer’s Disease (CERAD) test battery. The MCI group fulfilled research criteria for MCI and was limited to amnestic MCI as defined by a performance below −1.5 *SD* on the delayed recall trial of the CERAD word-list episodic memory tests. The DAT group fulfilled research criteria for DAT and had Mini-Mental Status Examination scores ≥ 18.

One CU group specifically included first-degree relatives of AD patients (*n* = 56). The other control group comprised *n* = 139 participants and was not enriched by study design. These two groups were recruited from the general public and were collapsed to form the CU group in the present study. Participants in this diagnostic group performed better than −1.5 *SD* below the German age, sex, and education-adjusted norms for the CERAD test battery and reported no subjective concerns about cognitive decline.

For all diagnostic groups, inclusion criteria were age ≥ 60 years, fluency in German, the ability to provide informed consent, and the availability of a study partner. Exclusion criteria included, but were not limited to, conditions that would prevent participation according to the study protocol (e.g., significant sensorimotor impairments), current or past diagnoses of psychiatric disorders (e.g., major depressive disorder, disorders due to psychoactive substance use), neurodegenerative disorders other than AD, vascular dementia, and a history of stroke with persistent clinical symptoms. The use of sedative, anticholinergic, and other anti-dementia medication as well as the use of investigational agents for treatment of cognitive impairment or dementia in all diagnostic groups except for the DAT group up to ≤ 1 month prior to screening and during the expected duration of the study led to exclusion.

## CSF sampling

CSF in DELCODE was collected following the same standard operation procedure at all study sites, with sampling in the morning after a minimum 12 hour fasting period. Lumbar puncture was performed by trained study staff using centrally packaged and distributed sampling material, using 15 ml polypropylene tubes for CSF collection. After centrifugation (10 min, RT, 2000 xG), CSF was aliquoted (0.5 ml matrix-coded storage vials, Thermo Fisher Scientific) and stored at –80 °C. CSF levels of Aβ_42_ and Aβ_40_ were determined using a commercially available kit according to vendor specifications (V-PLEX Aβ Peptide Panel 1 (6E10) Kit (K15200E), Mesoscale Diagnostics LLC, Rockville, USA).

## CSF amyloid positivity predictions

We closely followed an approach proposed by Hu *et al.* to predict cerebrospinal fluid (CSF)-based positivity for amyloid-β (Aβ) in individuals with missing CSF data.^1^ This analysis was performed in the whole DELCODE dataset and was not limited to sub-sample analysed in the present manuscript.

We fitted a binomial logistic regression model predicting CSF-based Aβ-positivity in participants with available CSF data and all required predictors (number of apolipoprotein E [APOE] ε4 alleles [0/1/2], APOE ε2 carriership [yes/no], age, plasma Aβ_42_/Aβ_40_ ratio, and plasma phosphorylated tau 181). These inclusion criteria resulted in a sample size of *n* = 328 (66 CU Aβ–, 20 CU Aβ+, 111 SCD Aβ–, 50 SCD Aβ+, 18 MCI Aβ–, 31 MCI Aβ+, 1 DAT Aβ–, 31 DAT Aβ+).

The model's explanatory power was substantial (Tjur's R^2^ = 0.60). Age (*b* = 0.09, *p* = 0.007), plasma phosphorylated tau 181 (*b* = 1.24, *p* < .001), plasma Aβ_42_/Aβ_40_ ratio (*b* = –113.19, *p* < .001), and having one copy of the APOE ε4 allele (*b* = 1.77, *p* < .001) were significant predictors in the model. Having two copy of the APOE ε4 allele (*b* = 18.00, p = 0.985) and being an APOE ε2 allele carrier (*b* = –0.13, *p* = 0.800) was not significantly associated with CSF Aβ positivity. The model’s discriminative ability was very high in the whole sample (AUC [95% C.I.] = 0.94 [0.91; 0.96]) and across diagnostic groups (AUC_CU_ = 0.94 [0.88; 1.00], AUC_SCD_ = 0.90 [0.85; 0.95], AUC_MCI_ = 0.94 [0.88; 1.00], AUC_DAT_ = 1.00 [*NA*; *NA*]). Note that the modelled C.I. for the DAT group was [1.00; 1.00] due to an AUC of 1.00. As perfect performance parameters are generally considered misleading, the C.I. is not reported. The original work by Hu *et al.* did not include plasma phosphorylated tau 181 as a predictor. We included this metric as this led to a substantial increase in model performance (AUC [95% C.I.]: 0.94 [0.91; 0.96] *versus* 0.91 [0.87; 0.94]).

The extracted subject-level probabilities ranged from 0 to 1, with higher values representing a higher probability of an individual being Aβ positive based on CSF Aβ_42_/Aβ_40_ ratios. The cut-off at 0.639 was identified using Youden’s criterion with a cost ratio of false negative to false positive classifications set to 1.5:1. This cut-off led to a sensitivity of 73.48%, a specificity of 94.90%, a positive predictive value of 90.65% and a negative predictive value of 84.16% in the whole sample. The trained model was then used to estimate the probability of CSF-based Aβ-positivity in all DELCODE participants with available predictors (*n* = 657; 201 CU, 308 SCD, 97 MCI, 69 DAT).

## Longitudinal segmentation of medial temporal lobe subregions

The longitudinal, template-based implementation of the ASHS algorithm^2^ relies on within-subject multivariate templates that were created using Advanced Normalization Tools (ANTs, ref.^3^). For T1-ASHS, these templates were calculated from all available T1-weighted images for a given participant, both at the original and an upsampled (0.5 × 0.5 × 1.0mm) resolution to approximate the anisotropic resolution of the T2-weighted images.^4^ For T2-ASHS, templates were generated from available T1- and T2-weighted images. ASHS was run on the resulting within-subject multivariate templates, using the atlases referenced in the main text. To obtain segmentations for each available time point, the resulting multilabel masks were warped onto the individual upsampled T1-weighted (for T1-ASHS) or original T2-weighted (for T2-ASHS) images using the warp fields and transformation matrices created during template calculation. All template segmentations underwent visual quality control and were manually edited if needed by experienced raters (H.B. and H.M.G).

# Supplementary results

## Pre-normalization of cognitive test scores

Using latent process modelling with subject-level random slopes and intercepts as well as spline-based link functions, we pre-normalized raw scores on the ADAS-Cog Delayed Word Recall (five knots with internal nodes at 2, 5, and 7), ADAS-Cog Figure Copying (four knots with internal nodes at 7 and 9), ADAS-Cog-13 (four quantile based knots), CDR-SB (six knots with internal nodes at 2, 5, 7, and 10), Clock Copying (five knots with internal nodes at 5, 7, and 9), Clock Drawing (five knots with internal nodes at 5, 7, and 9), FAQ (six knots with internal nodes at 2, 5, 10, 15), MMSE (six knots with internal nodes at 20, 24, 26, 28), and NPI-Q (six knots with internal nodes at 2, 5, 10, 15). This was done to ease LGCM fitting given the non-normal distributions (Supplementary Figure 7). These scores, along with the non-normalized raw scores from the remaining inventories, were then *z*-scored to participants in the CU Aβ–, CU Aβ+, and SCD Aβ+ groups and then passed latent growth curve models (LGCMs). These data are displayed in Supplementary Figure 8.

## Latent growth curve model fits

None of the fitted LGCMs failed to meet all of the *a priori* defined criteria for acceptable model fit. Three models slightly surpassed the threshold of RMSEA = 0.08 (hippocampal tail & PACC-5: RMSEA = 0.082; hippocampal tail & FCSRT-96: RMSEA = 0.080; inferior parietal cortex & PACC-5: RMSEA = 0.082). Given their acceptable CFIs (all > 0.96), these models were retained but should be interpreted cautiously.

With regards to variance structure, parahippocampal volumetric slopes did not consistently meet the critical *Z* threshold in all cases. However, as these violations were marginal and some models including this measure did show sufficient slope variance, we chose to include all models incorporating parahippocampal volumes, while advising readers to consider this limitation.

# References

1. Hu Y, Kirmess KM, Meyer MR, et al. Assessment of a Plasma Amyloid Probability Score to Estimate Amyloid Positron Emission Tomography Findings Among Adults With Cognitive Impairment. *Jama Netw Open*. 2022;5(4):e228392. doi:10.1001/jamanetworkopen.2022.8392

2. Yushkevich PA, Pluta JB, Wang H, et al. Automated Volumetry and Regional Thickness Analysis of Hippocampal Subfields and Medial Temporal Cortical Structures in Mild Cognitive Impairment. *Hum Brain Mapp*. 2015;36(1):258-287. doi:10.1002/hbm.22627

3. Avants B, Tustison NJ, Song G. Advanced Normalization Tools: V1.0. *Insight J*. Published online 2009. doi:10.54294/uvnhin

4. Xie L, Wisse LEM, Pluta J, et al. Automated segmentation of medial temporal lobe subregions on in vivo T1‐weighted MRI in early stages of Alzheimer’s disease. *Hum Brain Mapp*. 2019;40(12):3431-3451. doi:10.1002/hbm.24607

5. Filzmoser P, Gschwandtner M. mvoutlier: Multivariate Outlier Detection Based on Robust      Methods. Published online 2021. https://CRAN.R-project.org/package=mvoutlier

6. D’Orazio M. univOutl: Detection of Univariate Outliers. Published online 2022. https://CRAN.R-project.org/package=univOutl

7. Bates D, Mächler M, Bolker B, Walker S. Fitting Linear Mixed-Effects Models Using lme4. *J Stat Softw*. 2015;67(1):1-48. doi:10.18637/jss.v067.i01

#
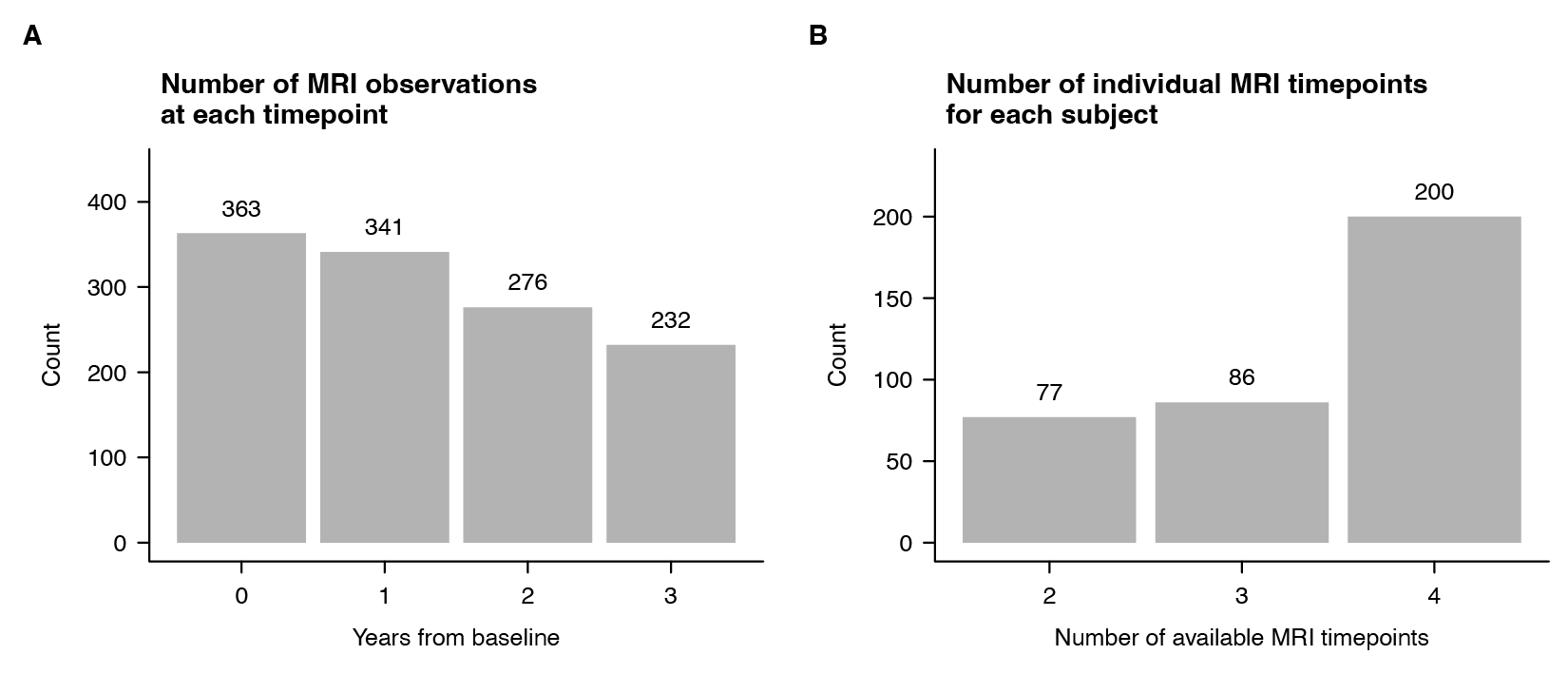
Supplementary figures

**Supplementary Figure 1 MRI data availability.** Bar plots indicate (**A**) the number of available scans by study time point and (**B**) the number of subjects with two, three, or four available scans.


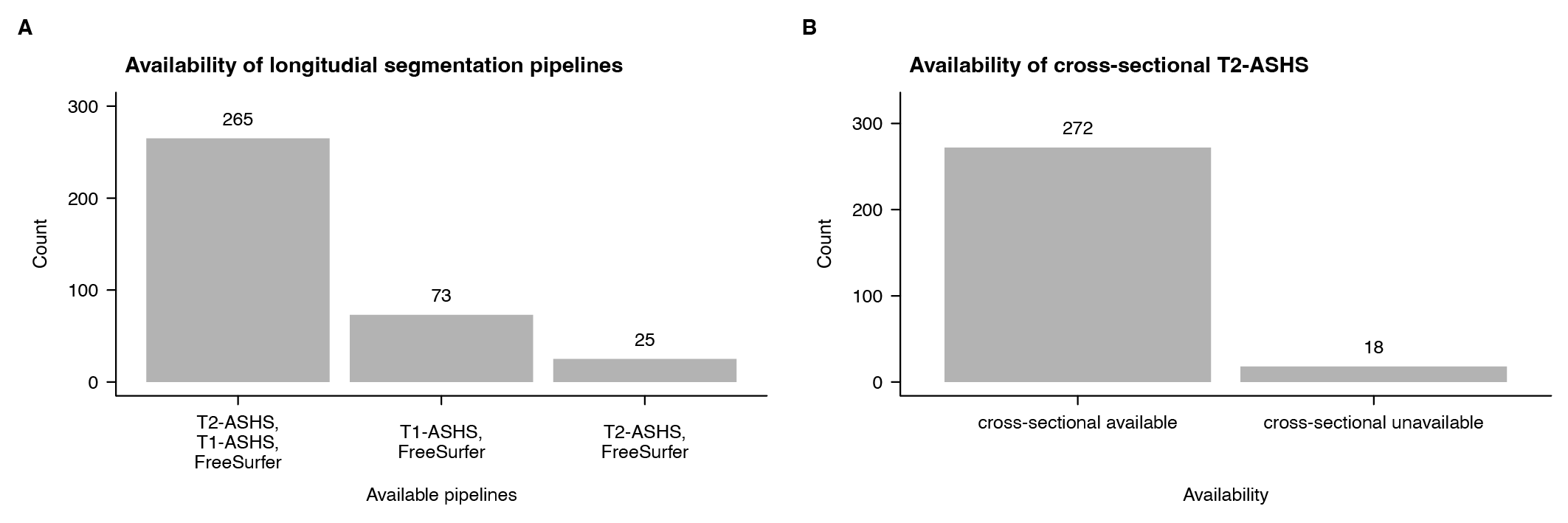


**Supplementary Figure 2 Segmentation pipeline availability.** Displayed are the counts of (**A**) different constellations of segmentation pipeline availability and (**B**) the availability of cross-sectional T2-ASHS in participants with available longitudinal T2-ASHS. The availability of either T1- or T2-ASHS were required for inclusion.


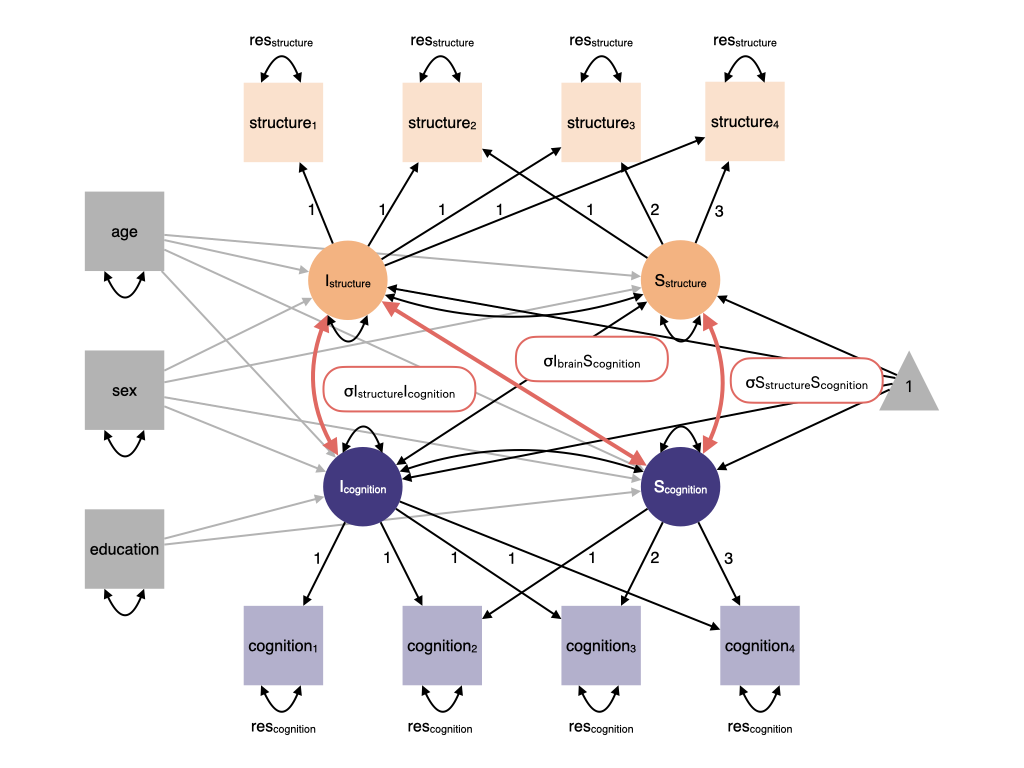


**Supplementary Figure 3 Bivariate latent growth curve model (LGCM) estimating the associations of longitudinally recorded measures of brain structure and cognition.** Manifest variables were recorded once (covariates) or four times (structure and cognitive variables). Parameters of interest were the covariance estimates representing baseline-baseline (σI_structure_I_cognition_), baseline-change (σI_structure_S_cognition_), and change-change (σS_structure_S_cognition_) associations of structure and cognition. Paths estimating the residual covariance between observed cognitive and brain variables at each time point are not shown for visual clarity. Paths labelled with numbers are fixed to those numbers. Paths labelled with the same name are constrained to be equal. Unlabelled paths are freely estimated.

Abbreviations: I, intercept; S, slope.


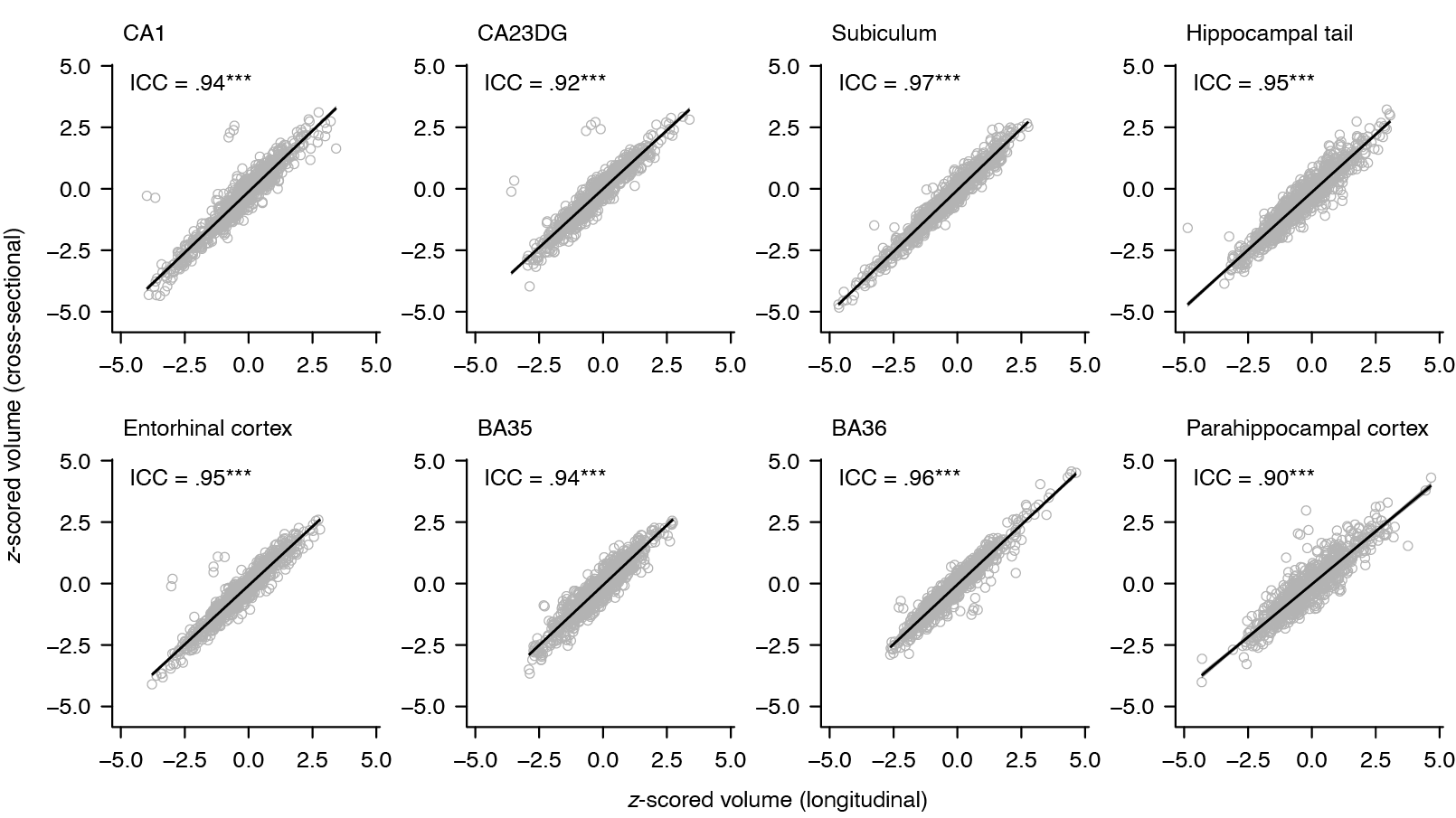


**Supplementary Figure 4 Scatter plots of baseline volumes obtained from the template-based and cross-sectional implementations of T2-ASHS.** Volumes were scaled and centred to the CU Aβ– group.

***p_FDR_ < .001. Abbreviations: ICC, intraclass correlation coefficient.


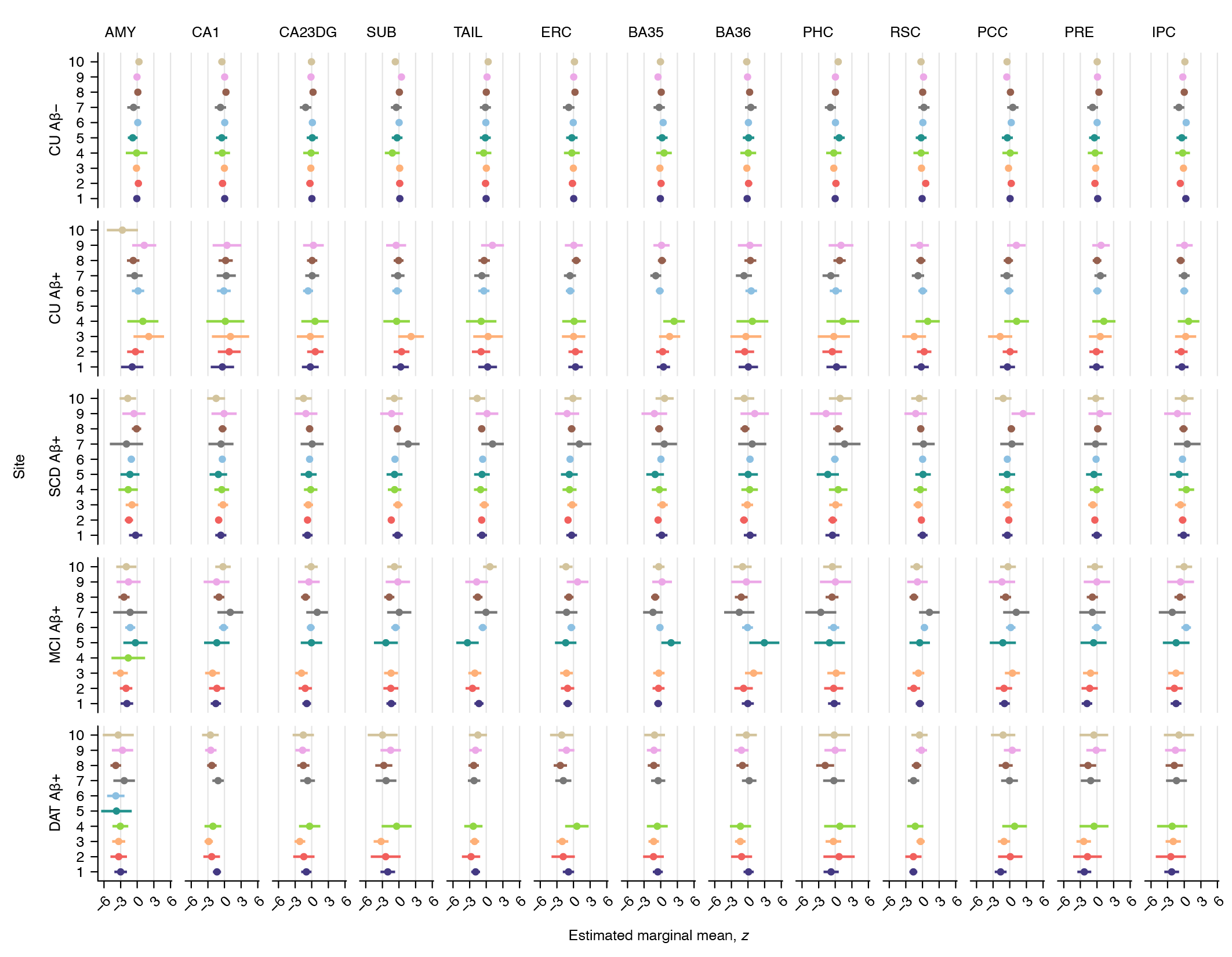


**Supplementary Figure 5 Estimated marginal means of the analysed structural MRI markers, stratified by clinical disease stage and DELCODE scanning site.** All markers were scaled and centred to the CU Aβ– group. Estimated marginal means are controlled for age and sex. Error bars denote 95% confidence intervals.

Abbreviations: Aβ, amyloid-β. AMY, amygdala. BA, Brodmann area. CA, cornu ammonis. CA23DG, cornu ammonis 2, 3, and dentate gyrus. CU, cognitively unimpaired. DAT, dementia of the Alzheimer type. ERC, entorhinal cortex. FDR, false discovery rate. IPC, inferior parietal cortex. MCI, mild cognitive impairment. PCC, posterior cingulate cortex. PHC, parahippocampal cortex. PRE, precuneus. RSC, retrosplenial cortex. SCD, subjective cognitive decline. SUB, subiculum. TAIL, hippocampal tail.


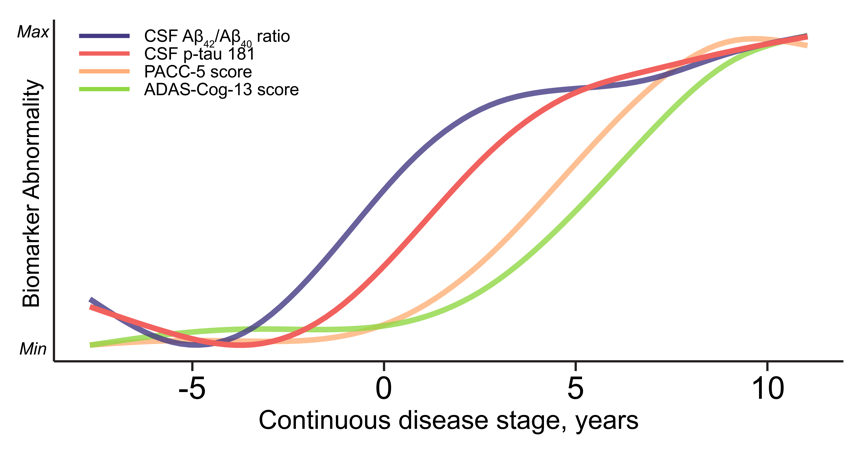


**Supplementary Figure 6 Biomarker trajectories across a continuous disease stage.** This model was modified from Lattmann-Grefe *et al.* by excluding structural MRI markers in order to prevent circular hypothesis testing in the current study.

Abbreviations: ADAS-Cog, Alzheimer’s Disease Assessment Scale-Cognitive subscale. PACC-5, Preclinical Alzheimer Cognitive Composite.


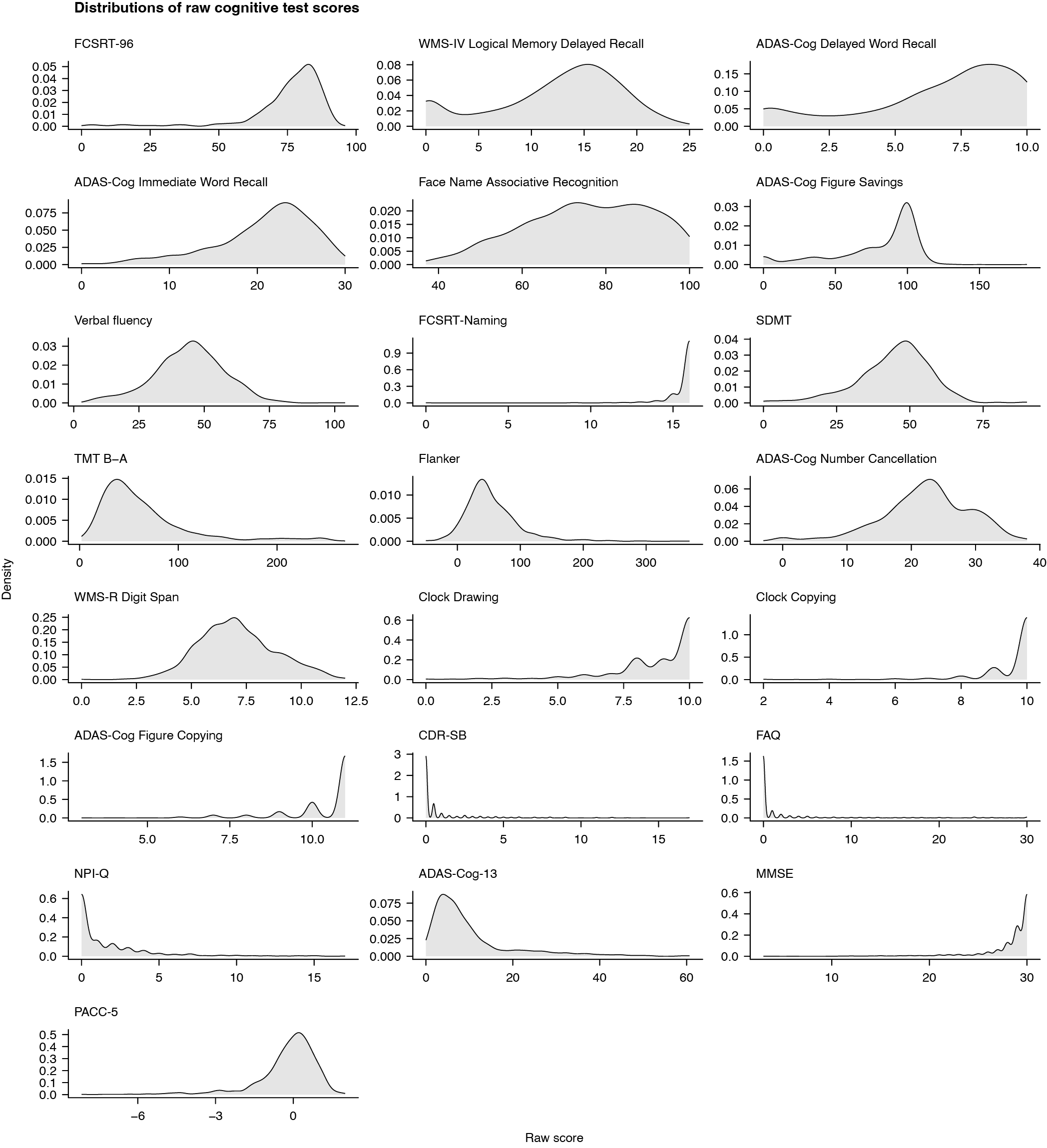


**Supplementary Figure 7 Density plots of all raw neuropsychological test scores.**

Abbreviations: ADAS-Cog, Alzheimer’s Disease Assessment Scale-Cognitive subscale. CSD-SB, Clinical Dementia Rating Sum of Boxes scale. FAQ, Functional Activities Questionnaire. FCSRT, Free and Cued Selective Reminding Test. MMSE, Mini-Mental State Examination. NPI-Q, Neuropsychiatric Inventory–Questionnaire. PACC-5, Preclinical Alzheimer Cognitive Composite. SDMT, Symbol-Digit Modalities Test. TMT, trail making test. WMS, Wechsler Memory Scale.


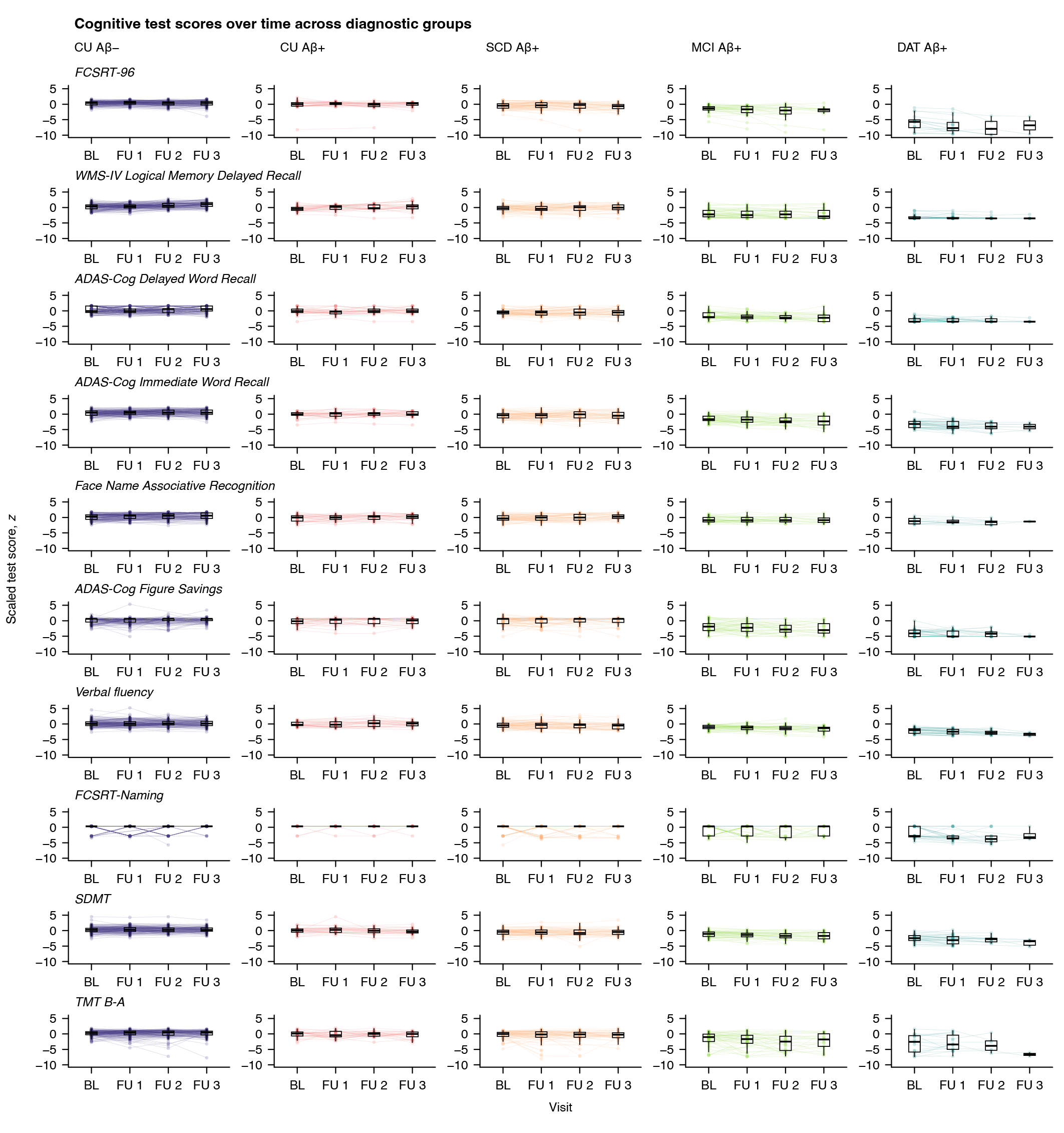


**Supplementary Figure 8 Standardized neuropsychological test scores by inventory, clinical disease stage, and visit.**

Abbreviations: ADAS-Cog, Alzheimer’s Disease Assessment Scale-Cognitive subscale. BL, baseline. CSD-SB, Clinical Dementia Rating Sum of Boxes scale. CU, cognitively unimpaired. DAT, dementia of the Alzheimer type. FAQ, Functional Activities Questionnaire. FCSRT, Free and Cued Selective Reminding Test. FU, follow-up. MCI, mild cognitive impairment. MMSE, Mini-Mental State Examination. NPI-Q, Neuropsychiatric Inventory–Questionnaire. PACC-5, Preclinical Alzheimer Cognitive Composite. SCD, subjective cognitive decline. SDMT, Symbol-Digit Modalities Test. TMT, trail making test. WMS, Wechsler Memory Scale.


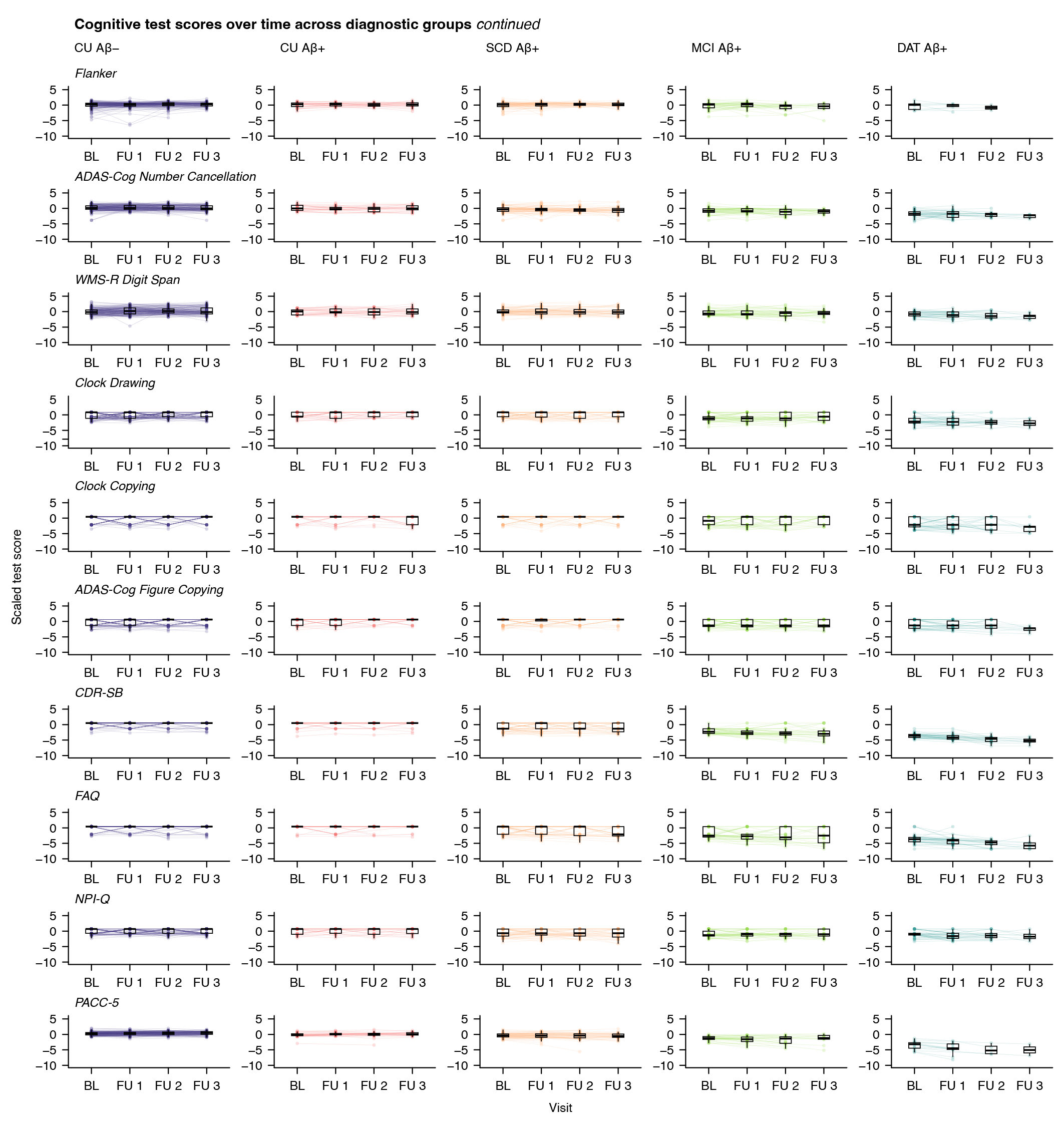


**Supplementary Figure 8** *continued*


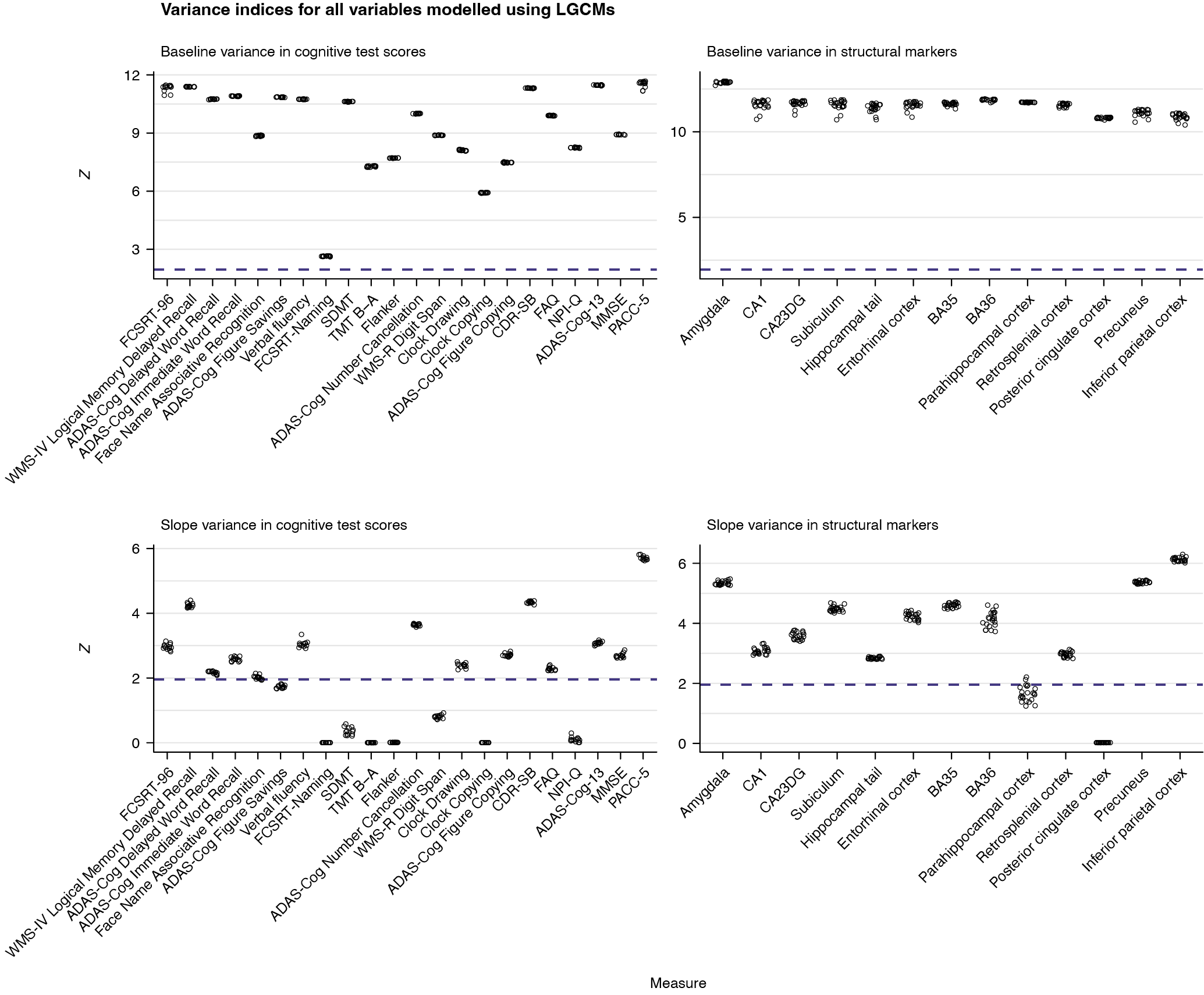
**Supplementary Figure 9 Variance indices for all atrophy markers and cognitive test scores included in the LGCM analysis.** The critical *Z* = 1.96 threshold is indicated by the dashed line. Measures falling below this threshold do not have sufficient variance to allow inference about covariance with other variables.

Abbreviations: ADAS-Cog, Alzheimer’s Disease Assessment Scale-Cognitive subscale. BA, Brodmann area. CA, cornu ammonis. CA23DG, cornu ammonis 2, 3, and dentate gyrus. CSD-SB, Clinical Dementia Rating Sum of Boxes scale. FAQ, Functional Activities Questionnaire. FCSRT, Free and Cued Selective Reminding Test. MMSE, Mini-Mental State Examination. NPI-Q, Neuropsychiatric Inventory–Questionnaire. PACC-5, Preclinical Alzheimer Cognitive Composite. TMT, Trail Making Test. WMS, Wechsler Memory Scale.


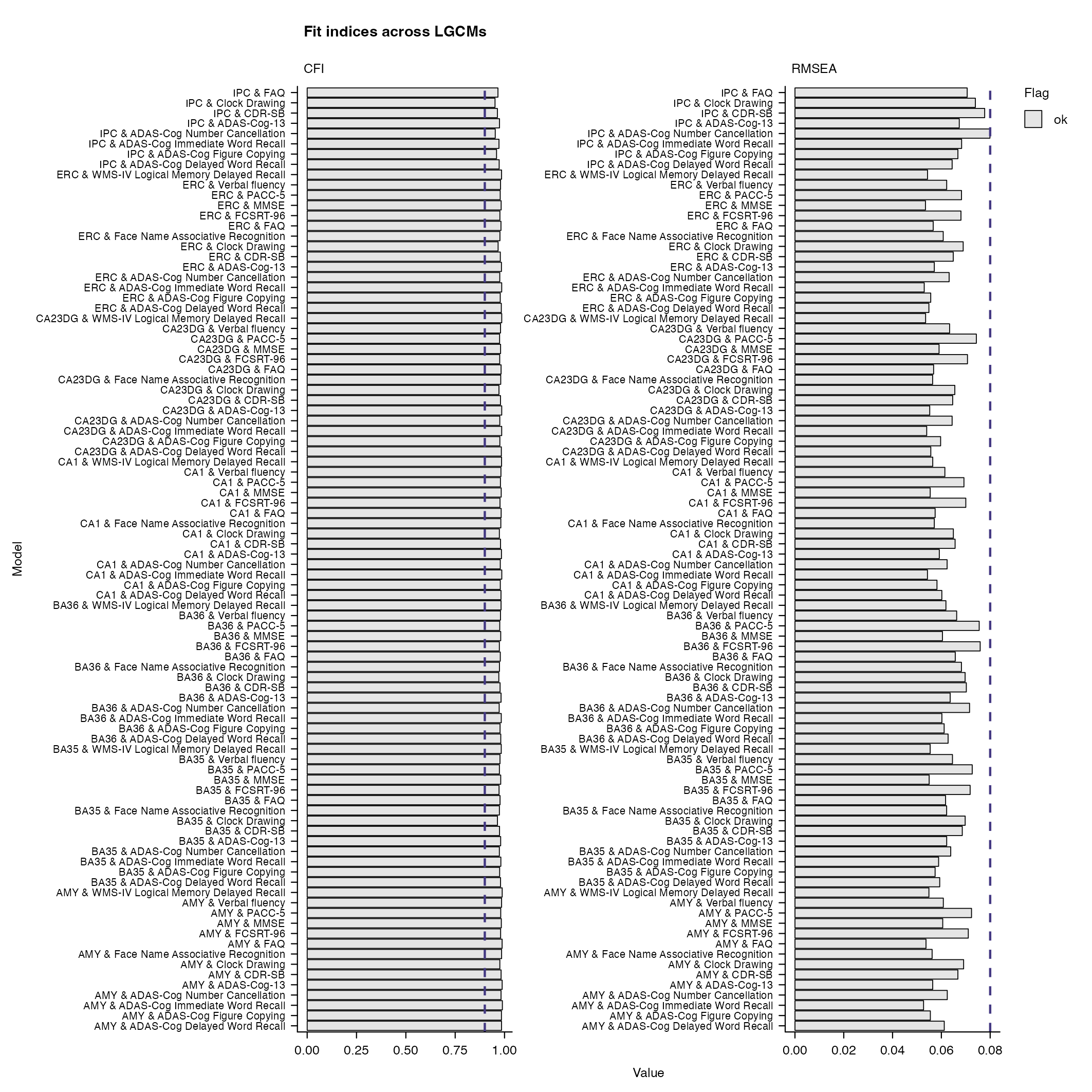


**Supplementary Figure 10 Fit indices for all LGCMs with sufficient variance in slopes and intercepts of both cognitive test scores and atrophy markers.** The critical values of RMSEA = 0.08 and CFI = 0.90 are indicated by the dashed lines.

Abbreviations: ADAS-Cog, Alzheimer’s Disease Assessment Scale-Cognitive subscale. BA, Brodmann area. CA, cornu ammonis. CA23DG, cornu ammonis 2, 3, and dentate gyrus. CFI, comparative fit index. CSD-SB, Clinical Dementia Rating Sum of Boxes scale. FAQ, Functional Activities Questionnaire. FCSRT, Free and Cued Selective Reminding Test. MMSE, Mini-Mental State Examination. PACC-5, Preclinical Alzheimer Cognitive Composite. RMSEA, root mean square error of approximation.


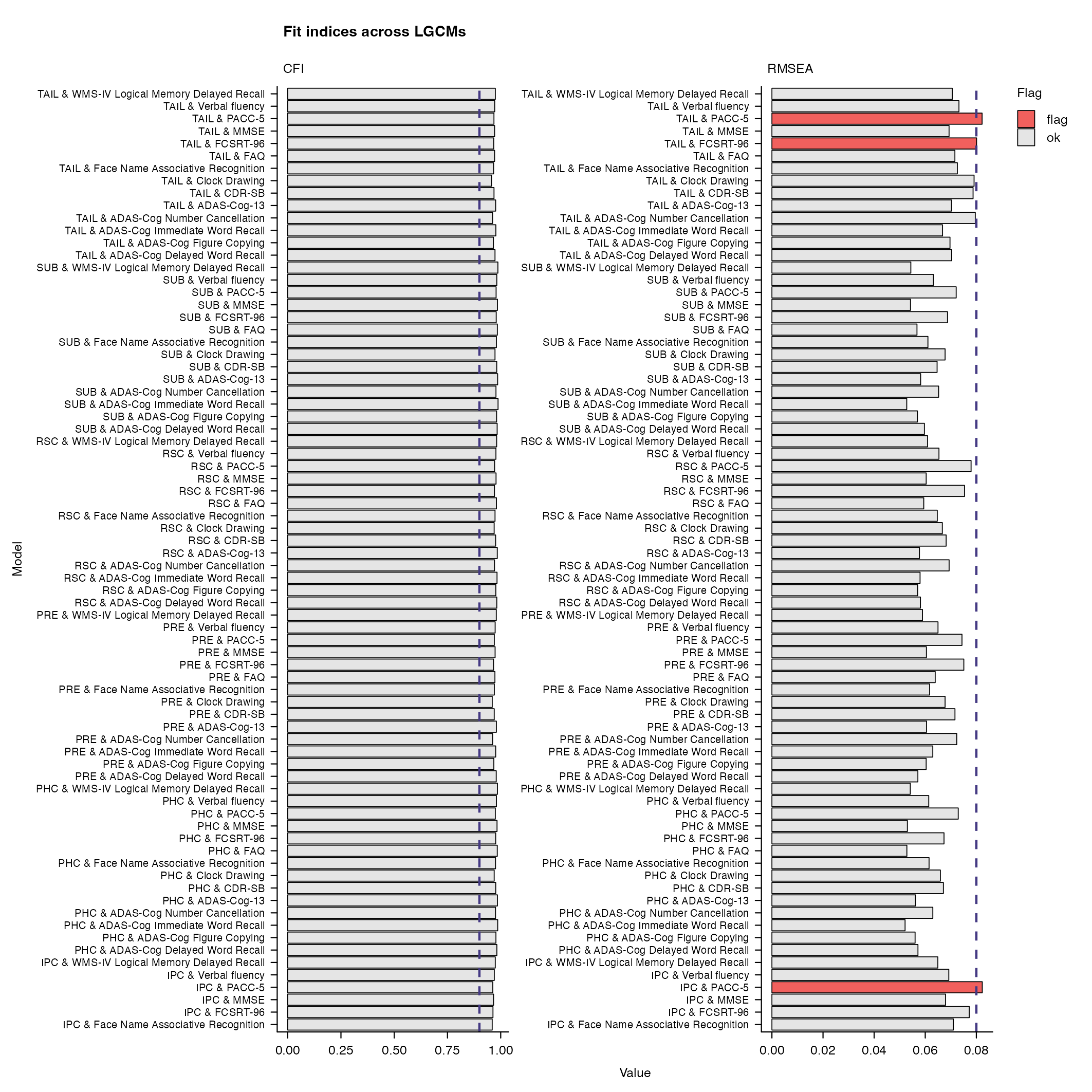


**Supplementary Figure 10** *continued*

# Supplementary tables

**Supplementary Table 1** Summary statistics for ANCOVA models estimating the effect of study site on scaled atrophy markers while controlling for participant age and sex, stratified by clinical disease stage.

| **Marker** | **Clinical disease stage** | **df** | **F** | ***p*_FDR_** |
| --- | --- | --- | --- | --- |
| Amygdala, volume | CU Aβ– | 9; 136 | 0.84 | .838 |
|  | CU Aβ+ | 8; 16 | 1.58 | .894 |
|  | SCD Aβ+ | 9; 64 | 1.12 | .924 |
|  | MCI Aβ+ | 9; 38 | 0.99 | .551 |
|  | DAT Aβ+ | 9; 25 | 0.46 | .953 |
| CA1, volume | CU Aβ– | 9; 139 | 0.97 | .765 |
|  | CU Aβ+ | 7; 18 | 0.23 | .973 |
|  | SCD Aβ+ | 9; 49 | 0.65 | .924 |
|  | MCI Aβ+ | 8; 21 | 1.67 | .357 |
|  | DAT Aβ+ | 7; 8 | 2.38 | .653 |
| CA23DG, volume | CU Aβ– | 9; 139 | 0.97 | .765 |
|  | CU Aβ+ | 7; 18 | 0.55 | .894 |
|  | SCD Aβ+ | 9; 49 | 0.48 | .924 |
|  | MCI Aβ+ | 8; 21 | 1.85 | .352 |
|  | DAT Aβ+ | 7; 8 | 1.02 | .792 |
| Subiculum, volume | CU Aβ– | 9; 139 | 1.51 | .765 |
|  | CU Aβ+ | 7; 18 | 0.90 | .894 |
|  | SCD Aβ+ | 9; 49 | 1.77 | .924 |
|  | MCI Aβ+ | 8; 21 | 1.13 | .497 |
|  | DAT Aβ+ | 7; 8 | 0.92 | .792 |
| Hippocampal tail, volume | CU Aβ– | 9; 139 | 0.28 | .980 |
|  | CU Aβ+ | 7; 18 | 0.59 | .894 |
|  | SCD Aβ+ | 9; 49 | 0.82 | .924 |
|  | MCI Aβ+ | 8; 21 | 3.02 | .131 |
|  | DAT Aβ+ | 7; 8 | 0.31 | .953 |
| Entorhinal cortex, volume | CU Aβ– | 9; 139 | 0.66 | .842 |
|  | CU Aβ+ | 7; 18 | 0.85 | .894 |
|  | SCD Aβ+ | 9; 49 | 0.71 | .924 |
|  | MCI Aβ+ | 8; 21 | 0.93 | .553 |
|  | DAT Aβ+ | 7; 8 | 1.64 | .653 |
| BA35, volume | CU Aβ– | 9; 139 | 1.11 | .765 |
|  | CU Aβ+ | 7; 18 | 2.13 | .894 |
|  | SCD Aβ+ | 9; 49 | 0.72 | .924 |
|  | MCI Aβ+ | 8; 21 | 1.55 | .371 |
|  | DAT Aβ+ | 7; 8 | 0.50 | .953 |
| BA36, volume | CU Aβ– | 9; 139 | 0.62 | .842 |
|  | CU Aβ+ | 7; 18 | 0.58 | .894 |
|  | SCD Aβ+ | 9; 49 | 1.10 | .924 |
|  | MCI Aβ+ | 8; 21 | 1.79 | .352 |
|  | DAT Aβ+ | 7; 8 | 1.76 | .653 |
| Parahippocampal cortex, volume | CU Aβ– | 9; 139 | 1.12 | .765 |
|  | CU Aβ+ | 7; 18 | 0.76 | .894 |
|  | SCD Aβ+ | 9; 49 | 1.01 | .924 |
|  | MCI Aβ+ | 8; 21 | 0.50 | .843 |
|  | DAT Aβ+ | 7; 8 | 0.90 | .792 |
| Retrosplenial cortex, average thickness | CU Aβ– | 9; 139 | 0.71 | .842 |
|  | CU Aβ+ | 7; 18 | 0.75 | .894 |
|  | SCD Aβ+ | 9; 49 | 0.59 | .924 |
|  | MCI Aβ+ | 8; 21 | 3.32 | .131 |
|  | DAT Aβ+ | 7; 8 | 2.20 | .653 |
| Posterior cingulate cortex, average thickness | CU Aβ– | 9; 139 | 1.32 | .765 |
|  | CU Aβ+ | 7; 18 | 1.13 | .894 |
|  | SCD Aβ+ | 9; 49 | 1.33 | .924 |
|  | MCI Aβ+ | 8; 21 | 1.13 | .497 |
|  | DAT Aβ+ | 7; 8 | 1.73 | .653 |
| Precuneus, average thickness | CU Aβ– | 9; 139 | 1.22 | .765 |
|  | CU Aβ+ | 7; 18 | 0.50 | .894 |
|  | SCD Aβ+ | 9; 49 | 0.47 | .924 |
|  | MCI Aβ+ | 8; 21 | 1.25 | .497 |
|  | DAT Aβ+ | 7; 8 | 1.38 | .714 |
| Inferior parietal cortex, average thickness | CU Aβ– | 9; 139 | 2.14 | .387 |
|  | CU Aβ+ | 7; 18 | 0.60 | .894 |
|  | SCD Aβ+ | 9; 49 | 0.41 | .924 |
|  | MCI Aβ+ | 8; 21 | 1.98 | .352 |
|  | DAT Aβ+ | 7; 8 | 0.26 | .953 |

FDR-correction was performed separately for each clinical disease stage across atrophy markers (13 comparisons per clinical disease stage). Abbreviations: CU, cognitively unimpaired. DAT, dementia of the Alzheimer type. df, degrees of freedom. MCI, mild cognitive impairment. SCD, subjective cognitive decline

**Supplementary Table 2** Summary statistics of ANCOVA models comparing baseline atrophy markers.

| **Marker** | **Type III sum of squares** | **df** | **Mean square** | ***F*** | ***p*** | ***p*_FDR_^†^** |
| --- | --- | --- | --- | --- | --- | --- |
| Amygdala, volume | 271.98 | 4; 331 | 67.99 | 44.73 | < .001*** | < .001*** |
| CA1, volume | 71.70 | 4; 283 | 17.92 | 16.13 | < .001*** | < .001*** |
| CA23DG, volume | 28.24 | 4; 283 | 7.06 | 7.34 | < .001*** | < .001*** |
| Subiculum, volume | 80.66 | 4; 283 | 20.16 | 20.11 | < .001*** | < .001*** |
| Hippocampal tail, volume | 60.06 | 4; 283 | 15.01 | 15.46 | < .001*** | < .001*** |
| Entorhinal cortex, volume | 40.25 | 4; 283 | 10.06 | 10.39 | < .001*** | < .001*** |
| BA35, volume | 12.84 | 4; 283 | 3.21 | 3.35 | .011* | .043* |
| BA36, volume | 7.99 | 4; 283 | 2.00 | 1.63 | .167 | .501 |
| Parahippocampal cortex, volume | 6.16 | 4; 283 | 1.54 | 1.19 | .317 | .506 |
| Retrosplenial cortex, average thickness | 14.82 | 4; 283 | 3.71 | 3.92 | .004** | .020* |
| Posterior cingulate cortex, average thickness | 5.56 | 4; 283 | 1.39 | 1.35 | .253 | .506 |
| Precuneus, average thickness | 45.70 | 4; 283 | 11.43 | 11.44 | < .001*** | < .001*** |
| Inferior parietal cortex, average thickness | 43.96 | 4; 283 | 10.99 | 10.40 | < .001*** | < .001*** |

All models controlled for age and sex. **p* < .05. ***p* < .01. *** *p* < .001. ^†^Values were FDR-corrected across regions (13 comparisons in total). Abbreviations: BA, Brodmann area. CA, cornu ammonis. CA23DG, cornu ammonis 2, 3, and dentate gyrus. df, degrees of freedom. FDR, false discovery rate. MS, mean squares.

**Supplementary Table 3** Summary statistics for planned *post hoc* comparisons of baseline atrophy markers between clinical disease stages.

| Marker | Contrast | Mean difference (*b*) [95% C.I.] | *SE* | *t* | *p*_raw_ | *p*_FDR_^†^ |
| --- | --- | --- | --- | --- | --- | --- |
| Amygdala, volume | CU Aβ+ – CU Aβ– | 0.07 [–0.62; 0.77] | 0.26 | 0.28 | .783 | .783 |
|  | SCD Aβ+ – CU Aβ– | –0.70 [–1.19; –0.21] | 0.19 | –3.75 | < .001*** | < .001*** |
|  | MCI Aβ+ – CU Aβ– | –1.57 [–2.13; –1.01] | 0.21 | –7.40 | < .001*** | < .001*** |
|  | DAT Aβ+ – CU Aβ– | –2.95 [–3.59; –2.31] | 0.24 | –12.21 | < .001*** | < .001*** |
|  | SCD Aβ+ – CU Aβ+ | –0.77 [–1.50; –0.04] | 0.28 | –2.78 | .006** | .007** |
|  | MCI Aβ+ – SCD Aβ+ | –0.87 [–1.47; –0.28] | 0.23 | –3.86 | < .001*** | < .001*** |
|  | DAT Aβ+ – MCI Aβ+ | –1.38 [–2.09; –0.67] | 0.27 | –5.16 | < .001*** | < .001*** |
| CA1, volume | CU Aβ+ – CU Aβ– | 0.36 [–0.23; 0.94] | 0.22 | 1.60 | .111 | .111 |
|  | SCD Aβ+ – CU Aβ– | –0.37 [–0.82; 0.09] | 0.17 | –2.13 | .034* | .047* |
|  | MCI Aβ+ – CU Aβ– | –0.81 [–1.37; –0.25] | 0.21 | –3.81 | < .001*** | < .001*** |
|  | DAT Aβ+ – CU Aβ– | –1.84 [–2.55; –1.13] | 0.27 | –6.84 | < .001*** | < .001*** |
|  | SCD Aβ+ – CU Aβ+ | –0.72 [–1.36; –0.08] | 0.24 | –2.99 | .003** | .005** |
|  | MCI Aβ+ – SCD Aβ+ | –0.44 [–1.06; 0.17] | 0.23 | –1.91 | .057 | .067 |
|  | DAT Aβ+ – MCI Aβ+ | –1.03 [–1.85; –0.21] | 0.31 | –3.32 | .001** | .002** |
| CA23DG, volume | CU Aβ+ – CU Aβ– | 0.00 [–0.54; 0.55] | 0.21 | 0.02 | .985 | .985 |
|  | SCD Aβ+ – CU Aβ– | –0.39 [–0.81; 0.04] | 0.16 | –2.42 | .016* | .038* |
|  | MCI Aβ+ – CU Aβ– | –0.59 [–1.11; –0.07] | 0.20 | –2.97 | .003** | .011* |
|  | DAT Aβ+ – CU Aβ– | –1.18 [–1.84; –0.52] | 0.25 | –4.70 | < .001*** | < .001*** |
|  | SCD Aβ+ – CU Aβ+ | –0.39 [–0.98; 0.20] | 0.22 | –1.74 | .083 | .116 |
|  | MCI Aβ+ – SCD Aβ+ | –0.20 [–0.77; 0.37] | 0.22 | –0.93 | .353 | .412 |
|  | DAT Aβ+ – MCI Aβ+ | –0.59 [–1.35; 0.17] | 0.29 | –2.04 | .042* | .074 |
| Subiculum, volume | CU Aβ+ – CU Aβ– | 0.17 [–0.39; 0.73] | 0.21 | 0.81 | .419 | .419 |
|  | SCD Aβ+ – CU Aβ– | –0.43 [–0.86; 0.00] | 0.16 | –2.65 | .009** | .013* |
|  | MCI Aβ+ – CU Aβ– | –0.91 [–1.44; –0.38] | 0.20 | –4.50 | < .001*** | < .001*** |
|  | DAT Aβ+ – CU Aβ– | –2.03 [–2.71; –1.35] | 0.26 | –7.94 | < .001*** | < .001*** |
|  | SCD Aβ+ – CU Aβ+ | –0.60 [–1.21; 0.00] | 0.23 | –2.63 | .009** | .013* |
|  | MCI Aβ+ – SCD Aβ+ | –0.48 [–1.06; 0.10] | 0.22 | –2.17 | .031* | .036* |
|  | DAT Aβ+ – MCI Aβ+ | –1.12 [–1.90; –0.34] | 0.30 | –3.80 | < .001*** | < .001*** |
| Hippocampal tail, volume | CU Aβ+ – CU Aβ– | –0.08 [–0.63; 0.47] | 0.21 | –0.38 | .703 | .703 |
|  | SCD Aβ+ – CU Aβ– | –0.37 [–0.79; 0.06] | 0.16 | –2.30 | .022* | .031* |
|  | MCI Aβ+ – CU Aβ– | –0.94 [–1.46; –0.41] | 0.20 | –4.72 | < .001*** | < .001*** |
|  | DAT Aβ+ – CU Aβ– | –1.73 [–2.40; –1.07] | 0.25 | –6.90 | < .001*** | < .001*** |
|  | SCD Aβ+ – CU Aβ+ | –0.29 [–0.89; 0.31] | 0.23 | –1.28 | .201 | .234 |
|  | MCI Aβ+ – SCD Aβ+ | –0.57 [–1.14; 0.00] | 0.22 | –2.63 | .009** | .016* |
|  | DAT Aβ+ – MCI Aβ+ | –0.80 [–1.56; –0.03] | 0.29 | –2.74 | .006** | .015* |
| Entorhinal cortex, volume | CU Aβ+ – CU Aβ– | 0.00 [–0.55; 0.55] | 0.21 | –0.01 | .995 | .995 |
|  | SCD Aβ+ – CU Aβ– | –0.44 [–0.86; –0.02] | 0.16 | –2.75 | .006** | .015* |
|  | MCI Aβ+ – CU Aβ– | –0.75 [–1.27; –0.22] | 0.20 | –3.76 | < .001*** | < .001*** |
|  | DAT Aβ+ – CU Aβ– | –1.39 [–2.05; –0.72] | 0.25 | –5.52 | < .001*** | < .001*** |
|  | SCD Aβ+ – CU Aβ+ | –0.44 [–1.03; 0.16] | 0.23 | –1.95 | .052 | .073 |
|  | MCI Aβ+ – SCD Aβ+ | –0.30 [–0.88; 0.27] | 0.22 | –1.41 | .160 | .187 |
|  | DAT Aβ+ – MCI Aβ+ | –0.64 [–1.41; 0.13] | 0.29 | –2.21 | .028* | .049* |
| BA35, volume | CU Aβ+ – CU Aβ– | 0.16 [–0.39; 0.70] | 0.21 | 0.77 | .441 | .618 |
|  | SCD Aβ+ – CU Aβ– | 0.05 [–0.37; 0.48] | 0.16 | 0.34 | .731 | .731 |
|  | MCI Aβ+ – CU Aβ– | –0.21 [–0.73; 0.31] | 0.20 | –1.07 | .287 | .502 |
|  | DAT Aβ+ – CU Aβ– | –0.80 [–1.46; –0.14] | 0.25 | –3.18 | .002** | .011* |
|  | SCD Aβ+ – CU Aβ+ | –0.10 [–0.70; 0.49] | 0.22 | –0.47 | .642 | .731 |
|  | MCI Aβ+ – SCD Aβ+ | –0.27 [–0.83; 0.30] | 0.22 | –1.23 | .218 | .502 |
|  | DAT Aβ+ – MCI Aβ+ | –0.58 [–1.35; 0.18] | 0.29 | –2.03 | .044* | .152 |
| Retrosplenial cortex, average thickness | CU Aβ+ – CU Aβ– | –0.12 [–0.66; 0.42] | 0.20 | –0.60 | .546 | .637 |
|  | SCD Aβ+ – CU Aβ– | –0.12 [–0.54; 0.30] | 0.16 | –0.76 | .448 | .627 |
|  | MCI Aβ+ – CU Aβ– | –0.45 [–0.97; 0.07] | 0.20 | –2.31 | .022* | .077 |
|  | DAT Aβ+ – CU Aβ– | –0.88 [–1.53; –0.22] | 0.25 | –3.55 | < .001*** | .003** |
|  | SCD Aβ+ – CU Aβ+ | 0.00 [–0.58; 0.59] | 0.22 | 0.01 | .988 | .988 |
|  | MCI Aβ+ – SCD Aβ+ | –0.33 [–0.90; 0.23] | 0.21 | –1.55 | .122 | .238 |
|  | DAT Aβ+ – MCI Aβ+ | –0.43 [–1.18; 0.33] | 0.29 | –1.50 | .136 | .238 |
| Precuneus, average thickness | CU Aβ+ – CU Aβ– | 0.40 [–0.15; 0.96] | 0.21 | 1.92 | .056 | .065 |
|  | SCD Aβ+ – CU Aβ– | –0.08 [–0.51; 0.35] | 0.16 | –0.50 | .618 | .618 |
|  | MCI Aβ+ – CU Aβ– | –0.62 [–1.15; –0.09] | 0.20 | –3.07 | .002** | .008** |
|  | DAT Aβ+ – CU Aβ– | –1.40 [–2.07; –0.73] | 0.26 | –5.49 | < .001*** | < .001*** |
|  | SCD Aβ+ – CU Aβ+ | –0.49 [–1.09; 0.12] | 0.23 | –2.12 | .035* | .048* |
|  | MCI Aβ+ – SCD Aβ+ | –0.54 [–1.12; 0.04] | 0.22 | –2.44 | .015* | .027* |
|  | DAT Aβ+ – MCI Aβ+ | –0.78 [–1.56; –0.01] | 0.29 | –2.66 | .008** | .019* |
| Inferior parietal cortex, average thickness | CU Aβ+ – CU Aβ– | 0.06 [–0.51; 0.63] | 0.22 | 0.28 | .776 | .776 |
|  | SCD Aβ+ – CU Aβ– | –0.06 [–0.50; 0.38] | 0.17 | –0.35 | .728 | .776 |
|  | MCI Aβ+ – CU Aβ– | –0.51 [–1.06; 0.04] | 0.21 | –2.45 | .015* | .035* |
|  | DAT Aβ+ – CU Aβ– | –1.57 [–2.27; –0.88] | 0.26 | –6.00 | < .001*** | < .001*** |
|  | SCD Aβ+ – CU Aβ+ | –0.12 [–0.74; 0.50] | 0.24 | –0.51 | .610 | .776 |
|  | MCI Aβ+ – SCD Aβ+ | –0.45 [–1.05; 0.15] | 0.23 | –1.99 | .048* | .084 |
|  | DAT Aβ+ – MCI Aβ+ | –1.07 [–1.87; –0.27] | 0.30 | –3.52 | < .001*** | .002** |

All models controlled for age and sex. **p* < .05. ***p* < .01. *** *p* < .001. **^†^**Values were FDR-corrected for contrasts within each marker (seven comparisons per marker). Abbreviations: BA, Brodmann area. CA, cornu ammonis. CA23DG, cornu ammonis 2, 3, and dentate gyrus. CU, cognitively unimpaired. DAT, dementia of the Alzheimer type. FDR, false discovery rate. MCI, mild cognitive impairment. SCD, subjective cognitive decline. SE, standard error.

**Supplementary Table 4** Summary statistics of the fixed effects in linear mixed effects models predicting structural MRI markers, derived from linear mixed effects models.

| **Marker** | **Predictor** | ***b* [95% C.I.]** | ***SE*** | ***t*** | ***df*** | ***η*^2^_partial_** | ***p*** |
| --- | --- | --- | --- | --- | --- | --- | --- |
| Amygdala, volume  *R*^2^_conditional_ = 0.99  *R*^2^_marginal_ = 0.46 | Intercept | 3.97 [2.32; 5.63] | 0.85 | 4.70 | 331.03 | 0.06 | < .001*** |
|  | Age, years | –0.06 [–0.08; –0.03] | 0.01 | –4.75 | 331.01 | 0.06 | < .001*** |
|  | Years since baseline | 0.12 [–0.05; 0.29] | 0.09 | 1.43 | 266.74 | 0.01 | .153 |
|  | Sex, male | 0.12 [–0.15; 0.39] | 0.14 | 0.89 | 330.92 | 0.00 | .372 |
|  | HC Aβ+ diagnosis | 0.05 [–0.47; 0.56] | 0.26 | 0.18 | 330.43 | 0.00 | .857 |
|  | SCD Aβ+ diagnosis | –0.71 [–1.07; –0.34] | 0.19 | –3.81 | 330.93 | 0.04 | < .001*** |
|  | MCI Aβ+ diagnosis | –1.60 [–2.01; –1.18] | 0.21 | –7.55 | 331.02 | 0.15 | < .001*** |
|  | DAT Aβ+ diagnosis | –2.99 [–3.47; –2.52] | 0.24 | –12.42 | 331.55 | 0.32 | < .001*** |
|  | Age, years ⨉ years since baseline | 0.00 [0.00; 0.00] | 0.00 | –1.87 | 266.73 | 0.01 | .063 |
|  | Sex, male ⨉ years since baseline | –0.01 [–0.04; 0.02] | 0.01 | –0.62 | 257.62 | 0.00 | .538 |
|  | HC Aβ+ diagnosis ⨉ years since baseline | –0.07 [–0.12; –0.03] | 0.02 | –2.99 | 224.83 | 0.04 | .003** |
|  | SCD Aβ+ diagnosis ⨉ years since baseline | –0.05 [–0.09; –0.01] | 0.02 | –2.70 | 254.74 | 0.03 | .007** |
|  | MCI Aβ+ diagnosis ⨉ years since baseline | –0.11 [–0.16; –0.07] | 0.02 | –5.19 | 272.70 | 0.09 | < .001*** |
|  | DAT Aβ+ diagnosis ⨉ years since baseline | –0.14 [–0.19; –0.08] | 0.03 | –4.90 | 347.63 | 0.06 | < .001*** |
| CA1, volume  *R*^2^_conditional_ = 0.98  *R*^2^_marginal_ = 0.27 | Intercept | 2.75 [1.23; 4.27] | 0.78 | 3.55 | 283.05 | 0.04 | < .001*** |
|  | Age, years | –0.04 [–0.06; –0.02] | 0.01 | –3.51 | 283.06 | 0.04 | < .001*** |
|  | Years since baseline | 0.13 [–0.02; 0.28] | 0.08 | 1.64 | 257.78 | 0.01 | .102 |
|  | Sex, male | –0.06 [–0.32; 0.19] | 0.13 | –0.50 | 282.87 | 0.00 | .620 |
|  | HC Aβ+ diagnosis | 0.35 [–0.08; 0.79] | 0.22 | 1.60 | 282.61 | 0.01 | .110 |
|  | SCD Aβ+ diagnosis | –0.37 [–0.71; –0.04] | 0.17 | –2.17 | 282.95 | 0.02 | .031* |
|  | MCI Aβ+ diagnosis | –0.83 [–1.25; –0.42] | 0.21 | –3.94 | 283.17 | 0.05 | < .001*** |
|  | DAT Aβ+ diagnosis | –1.88 [–2.40; –1.35] | 0.27 | –7.01 | 283.47 | 0.15 | < .001*** |
|  | Age, years ⨉ years since baseline | 0.00 [0.00; 0.00] | 0.00 | –1.83 | 257.06 | 0.01 | .069 |
|  | Sex, male ⨉ years since baseline | 0.00 [–0.03; 0.02] | 0.01 | –0.27 | 246.36 | 0.00 | .787 |
|  | HC Aβ+ diagnosis ⨉ years since baseline | –0.02 [–0.06; 0.02] | 0.02 | –1.11 | 229.85 | 0.01 | .269 |
|  | SCD Aβ+ diagnosis ⨉ years since baseline | –0.02 [–0.05; 0.02] | 0.02 | –0.99 | 252.49 | 0.00 | .325 |
|  | MCI Aβ+ diagnosis ⨉ years since baseline | –0.05 [–0.09; 0.00] | 0.02 | –2.13 | 280.11 | 0.02 | .034* |
|  | DAT Aβ+ diagnosis ⨉ years since baseline | –0.08 [–0.13; –0.02] | 0.03 | –2.63 | 319.87 | 0.02 | .009** |
| CA23DG, volume  *R*^2^_conditional_ = 0.98  *R*^2^_marginal_ = 0.18 | Intercept | 1.89 [0.47; 3.32] | 0.73 | 2.60 | 282.85 | 0.02 | .010** |
|  | Age, years | –0.03 [–0.05; –0.01] | 0.01 | –2.53 | 282.85 | 0.02 | .012* |
|  | Years since baseline | 0.10 [–0.05; 0.25] | 0.08 | 1.33 | 251.68 | 0.01 | .185 |
|  | Sex, male | –0.15 [–0.38; 0.09] | 0.12 | –1.23 | 282.67 | 0.01 | .221 |
|  | HC Aβ+ diagnosis | –0.02 [–0.43; 0.39] | 0.21 | –0.09 | 282.38 | 0.00 | .926 |
|  | SCD Aβ+ diagnosis | –0.40 [–0.71; –0.08] | 0.16 | –2.47 | 282.74 | 0.02 | .014* |
|  | MCI Aβ+ diagnosis | –0.59 [–0.98; –0.20] | 0.20 | –2.97 | 282.98 | 0.03 | .003** |
|  | DAT Aβ+ diagnosis | –1.21 [–1.70; –0.71] | 0.25 | –4.81 | 283.30 | 0.08 | < .001*** |
|  | Age, years ⨉ years since baseline | 0.00 [0.00; 0.00] | 0.00 | –2.19 | 250.99 | 0.02 | .029* |
|  | Sex, male ⨉ years since baseline | 0.00 [–0.02; 0.02] | 0.01 | –0.07 | 240.30 | 0.00 | .946 |
|  | HC Aβ+ diagnosis ⨉ years since baseline | –0.01 [–0.04; 0.03] | 0.02 | –0.28 | 223.93 | 0.00 | .783 |
|  | SCD Aβ+ diagnosis ⨉ years since baseline | –0.03 [–0.06; 0.00] | 0.02 | –1.86 | 246.51 | 0.01 | .064 |
|  | MCI Aβ+ diagnosis ⨉ years since baseline | –0.05 [–0.09; –0.01] | 0.02 | –2.30 | 273.51 | 0.02 | .022* |
|  | DAT Aβ+ diagnosis ⨉ years since baseline | –0.10 [–0.15; –0.04] | 0.03 | –3.50 | 313.44 | 0.04 | < .001*** |
| Subiculum, volume  *R*^2^_conditional_ = 0.99  *R*^2^_marginal_ = 0.37 | Intercept | 4.39 [2.95; 5.83] | 0.74 | 5.96 | 283.27 | 0.11 | < .001*** |
|  | Age, years | –0.07 [–0.09; –0.04] | 0.01 | –6.04 | 283.27 | 0.11 | < .001*** |
|  | Years since baseline | 0.17 [0.02; 0.32] | 0.08 | 2.19 | 252.45 | 0.02 | .029* |
|  | Sex, male | 0.19 [–0.05; 0.43] | 0.12 | 1.58 | 283.09 | 0.01 | .114 |
|  | HC Aβ+ diagnosis | 0.16 [–0.25; 0.58] | 0.21 | 0.78 | 282.82 | 0.00 | .435 |
|  | SCD Aβ+ diagnosis | –0.44 [–0.76; –0.12] | 0.16 | –2.70 | 283.17 | 0.03 | .007** |
|  | MCI Aβ+ diagnosis | –0.91 [–1.31; –0.52] | 0.20 | –4.55 | 283.39 | 0.07 | < .001*** |
|  | DAT Aβ+ diagnosis | –2.04 [–2.54; –1.54] | 0.25 | –8.02 | 283.70 | 0.18 | < .001*** |
|  | Age, years ⨉ years since baseline | 0.00 [–0.01; 0.00] | 0.00 | –2.67 | 251.85 | 0.03 | .008** |
|  | Sex, male ⨉ years since baseline | –0.01 [–0.03; 0.02] | 0.01 | –0.74 | 241.00 | 0.00 | .459 |
|  | HC Aβ+ diagnosis ⨉ years since baseline | –0.02 [–0.06; 0.03] | 0.02 | –0.74 | 224.70 | 0.00 | .463 |
|  | SCD Aβ+ diagnosis ⨉ years since baseline | –0.05 [–0.08; –0.02] | 0.02 | –2.91 | 247.55 | 0.03 | .004** |
|  | MCI Aβ+ diagnosis ⨉ years since baseline | –0.07 [–0.11; –0.03] | 0.02 | –3.13 | 273.29 | 0.03 | .002** |
|  | DAT Aβ+ diagnosis ⨉ years since baseline | –0.12 [–0.17; –0.06] | 0.03 | –3.93 | 313.12 | 0.05 | < .001*** |
| Hippocampal tail, volume  *R*^2^_conditional_ = 0.97  *R*^2^_marginal_ = 0.35 | Intercept | 4.49 [3.08; 5.91] | 0.72 | 6.22 | 283.33 | 0.12 | < .001*** |
|  | Age, years | –0.07 [–0.09; –0.04] | 0.01 | –6.21 | 283.35 | 0.12 | < .001*** |
|  | Years since baseline | 0.14 [–0.06; 0.34] | 0.10 | 1.39 | 247.63 | 0.01 | .165 |
|  | Sex, male | –0.04 [–0.27; 0.19] | 0.12 | –0.34 | 282.99 | 0.00 | .738 |
|  | HC Aβ+ diagnosis | –0.09 [–0.49; 0.31] | 0.21 | –0.44 | 282.48 | 0.00 | .660 |
|  | SCD Aβ+ diagnosis | –0.35 [–0.67; –0.04] | 0.16 | –2.22 | 283.14 | 0.02 | .027* |
|  | MCI Aβ+ diagnosis | –0.95 [–1.34; –0.57] | 0.20 | –4.83 | 283.56 | 0.08 | < .001*** |
|  | DAT Aβ+ diagnosis | –1.77 [–2.26; –1.28] | 0.25 | –7.11 | 284.16 | 0.15 | < .001*** |
|  | Age, years ⨉ years since baseline | 0.00 [–0.01; 0.00] | 0.00 | –1.57 | 246.97 | 0.01 | .119 |
|  | Sex, male ⨉ years since baseline | –0.02 [–0.05; 0.01] | 0.02 | –1.05 | 236.29 | 0.00 | .293 |
|  | HC Aβ+ diagnosis ⨉ years since baseline | –0.01 [–0.07; 0.04] | 0.03 | –0.50 | 220.04 | 0.00 | .614 |
|  | SCD Aβ+ diagnosis ⨉ years since baseline | –0.05 [–0.09; 0.00] | 0.02 | –2.12 | 242.57 | 0.02 | .035* |
|  | MCI Aβ+ diagnosis ⨉ years since baseline | –0.03 [–0.09; 0.02] | 0.03 | –1.12 | 269.10 | 0.00 | .263 |
|  | DAT Aβ+ diagnosis ⨉ years since baseline | –0.04 [–0.12; 0.03] | 0.04 | –1.09 | 308.93 | 0.00 | .276 |
| Entorhinal cortex, volume  *R*^2^_conditional_ = 0.97, *R*^2^_marginal_ = 0.24 | Intercept | 2.23 [0.81; 3.66] | 0.72 | 3.08 | 283.23 | 0.03 | .002** |
|  | Age, years | –0.03 [–0.05; –0.01] | 0.01 | –3.10 | 283.24 | 0.03 | .002** |
|  | Years since baseline | 0.25 [0.08; 0.43] | 0.09 | 2.91 | 253.61 | 0.03 | .004** |
|  | Sex, male | 0.08 [–0.16; 0.31] | 0.12 | 0.65 | 282.98 | 0.00 | .515 |
|  | HC Aβ+ diagnosis | –0.03 [–0.44; 0.37] | 0.21 | –0.17 | 282.60 | 0.00 | .866 |
|  | SCD Aβ+ diagnosis | –0.47 [–0.78; –0.15] | 0.16 | –2.92 | 283.09 | 0.03 | .004** |
|  | MCI Aβ+ diagnosis | –0.77 [–1.16; –0.39] | 0.20 | –3.91 | 283.40 | 0.05 | < .001*** |
|  | DAT Aβ+ diagnosis | –1.42 [–1.91; –0.92] | 0.25 | –5.65 | 283.83 | 0.10 | < .001*** |
|  | Age, years ⨉ years since baseline | 0.00 [–0.01; 0.00] | 0.00 | –2.94 | 252.92 | 0.03 | .004** |
|  | Sex, male ⨉ years since baseline | –0.01 [–0.03; 0.02] | 0.01 | –0.45 | 242.20 | 0.00 | .653 |
|  | HC Aβ+ diagnosis ⨉ years since baseline | –0.07 [–0.12; –0.02] | 0.02 | –3.01 | 225.81 | 0.04 | .003** |
|  | SCD Aβ+ diagnosis ⨉ years since baseline | –0.05 [–0.08; –0.01] | 0.02 | –2.42 | 248.46 | 0.02 | .016* |
|  | MCI Aβ+ diagnosis ⨉ years since baseline | –0.08 [–0.13; –0.03] | 0.02 | –3.30 | 275.28 | 0.04 | .001** |
|  | DAT Aβ+ diagnosis ⨉ years since baseline | –0.17 [–0.24; –0.11] | 0.03 | –5.24 | 315.30 | 0.08 | < .001*** |
| BA35, volume  *R*^2^_conditional_ = 0.97  *R*^2^_marginal_ = 0.13 | Intercept | 2.43 [1.01; 3.86] | 0.73 | 3.35 | 283.14 | 0.04 | < .001*** |
|  | Age, years | –0.04 [–0.06; –0.02] | 0.01 | –3.41 | 283.15 | 0.04 | < .001*** |
|  | Years since baseline | 0.19 [0.01; 0.37] | 0.09 | 2.02 | 258.01 | 0.02 | .044* |
|  | Sex, male | 0.15 [–0.09; 0.38] | 0.12 | 1.22 | 282.88 | 0.01 | .224 |
|  | HC Aβ+ diagnosis | 0.15 [–0.26; 0.55] | 0.21 | 0.71 | 282.50 | 0.00 | .478 |
|  | SCD Aβ+ diagnosis | 0.05 [–0.26; 0.37] | 0.16 | 0.33 | 283.00 | 0.00 | .745 |
|  | MCI Aβ+ diagnosis | –0.22 [–0.61; 0.17] | 0.20 | –1.11 | 283.32 | 0.00 | .268 |
|  | DAT Aβ+ diagnosis | –0.82 [–1.31; –0.33] | 0.25 | –3.28 | 283.76 | 0.04 | .001** |
|  | Age, years ⨉ years since baseline | 0.00 [–0.01; 0.00] | 0.00 | –2.29 | 257.43 | 0.02 | .023* |
|  | Sex, male ⨉ years since baseline | –0.01 [–0.04; 0.02] | 0.01 | –0.39 | 246.40 | 0.00 | .695 |
|  | HC Aβ+ diagnosis ⨉ years since baseline | –0.07 [–0.12; –0.02] | 0.02 | –2.73 | 229.91 | 0.03 | .007** |
|  | SCD Aβ+ diagnosis ⨉ years since baseline | –0.04 [–0.08; 0.00] | 0.02 | –2.04 | 253.17 | 0.02 | .043* |
|  | MCI Aβ+ diagnosis ⨉ years since baseline | –0.04 [–0.09; 0.01] | 0.03 | –1.69 | 278.95 | 0.01 | .093 |
|  | DAT Aβ+ diagnosis ⨉ years since baseline | –0.16 [–0.22; –0.09] | 0.03 | –4.49 | 318.92 | 0.06 | < .001*** |
| BA36, volume  *R*^2^_conditional_ = 0.99  *R*^2^_marginal_ = 0.07 | Intercept | 1.81 [0.21; 3.42] | 0.82 | 2.21 | 283.03 | 0.02 | .028* |
|  | Age, years | –0.03 [–0.05; 0.00] | 0.01 | –2.28 | 283.03 | 0.02 | .023* |
|  | Years since baseline | 0.07 [–0.05; 0.20] | 0.06 | 1.13 | 223.34 | 0.01 | .259 |
|  | Sex, male | 0.17 [–0.09; 0.44] | 0.14 | 1.27 | 282.93 | 0.01 | .204 |
|  | HC Aβ+ diagnosis | 0.17 [–0.29; 0.63] | 0.23 | 0.72 | 282.77 | 0.00 | .473 |
|  | SCD Aβ+ diagnosis | 0.05 [–0.31; 0.40] | 0.18 | 0.25 | 282.97 | 0.00 | .799 |
|  | MCI Aβ+ diagnosis | –0.14 [–0.58; 0.29] | 0.22 | –0.65 | 283.10 | 0.00 | .519 |
|  | DAT Aβ+ diagnosis | –0.62 [–1.18; –0.07] | 0.28 | –2.19 | 283.28 | 0.02 | .029* |
|  | Age, years ⨉ years since baseline | 0.00 [0.00; 0.00] | 0.00 | –1.47 | 222.70 | 0.01 | .142 |
|  | Sex, male ⨉ years since baseline | 0.00 [–0.02; 0.02] | 0.01 | –0.04 | 212.59 | 0.00 | .968 |
|  | HC Aβ+ diagnosis ⨉ years since baseline | –0.03 [–0.06; 0.00] | 0.02 | –1.76 | 197.24 | 0.02 | .080 |
|  | SCD Aβ+ diagnosis ⨉ years since baseline | –0.03 [–0.06; –0.01] | 0.01 | –2.37 | 218.48 | 0.03 | .019* |
|  | MCI Aβ+ diagnosis ⨉ years since baseline | –0.04 [–0.08; –0.01] | 0.02 | –2.50 | 244.15 | 0.03 | .013* |
|  | DAT Aβ+ diagnosis ⨉ years since baseline | –0.18 [–0.23; –0.13] | 0.02 | –7.51 | 282.73 | 0.17 | < .001*** |
| Parahippocampal cortex, volume^†^  *R*^2^_conditional_ = 0.97  *R*^2^_marginal_ = 0.07 | Intercept | 2.22 [0.57; 3.87] | 0.84 | 2.63 | 283.35 | 0.02 | .009** |
|  | Age, years | –0.03 [–0.06; –0.01] | 0.01 | –2.68 | 283.36 | 0.02 | .008** |
|  | Years since baseline | 0.05 [–0.12; 0.22] | 0.09 | 0.61 | 217.77 | 0.00 | .543 |
|  | Sex, male | 0.15 [–0.13; 0.42] | 0.14 | 1.06 | 283.13 | 0.00 | .289 |
|  | HC Aβ+ diagnosis | 0.26 [–0.22; 0.73] | 0.24 | 1.06 | 282.79 | 0.00 | .288 |
|  | SCD Aβ+ diagnosis | –0.01 [–0.37; 0.36] | 0.19 | –0.05 | 283.22 | 0.00 | .961 |
|  | MCI Aβ+ diagnosis | –0.29 [–0.74; 0.16] | 0.23 | –1.26 | 283.50 | 0.01 | .208 |
|  | DAT Aβ+ diagnosis | –0.34 [–0.91; 0.23] | 0.29 | –1.16 | 283.88 | 0.00 | .246 |
|  | Age, years ⨉ years since baseline | 0.00 [0.00; 0.00] | 0.00 | –1.16 | 217.15 | 0.01 | .249 |
|  | Sex, male ⨉ years since baseline | –0.02 [–0.05; 0.01] | 0.01 | –1.44 | 207.41 | 0.01 | .151 |
|  | HC Aβ+ diagnosis ⨉ years since baseline | 0.03 [–0.02; 0.07] | 0.02 | 1.17 | 192.71 | 0.01 | .243 |
|  | SCD Aβ+ diagnosis ⨉ years since baseline | –0.01 [–0.05; 0.03] | 0.02 | –0.49 | 213.02 | 0.00 | .624 |
|  | MCI Aβ+ diagnosis ⨉ years since baseline | –0.03 [–0.08; 0.01] | 0.02 | –1.44 | 240.06 | 0.01 | .151 |
|  | DAT Aβ+ diagnosis ⨉ years since baseline | –0.14 [–0.21; –0.08] | 0.03 | –4.32 | 274.19 | 0.06 | < .001*** |
| Retrosplenial cortex, average thickness  *R*^2^_conditional_ = 0.96  *R*^2^_marginal_ = 0.14 | Intercept | 2.39 [0.98; 3.81] | 0.72 | 3.31 | 283.23 | 0.04 | .001** |
|  | Age, years | –0.04 [–0.06; –0.01] | 0.01 | –3.32 | 283.23 | 0.04 | .001** |
|  | Years since baseline | 0.03 [–0.14; 0.20] | 0.09 | 0.34 | 251.39 | 0.00 | .734 |
|  | Sex, male | –0.01 [–0.24; 0.23] | 0.12 | –0.04 | 282.99 | 0.00 | .965 |
|  | HC Aβ+ diagnosis | –0.14 [–0.55; 0.26] | 0.21 | –0.70 | 282.54 | 0.00 | .483 |
|  | SCD Aβ+ diagnosis | –0.08 [–0.40; 0.23] | 0.16 | –0.53 | 283.22 | 0.00 | .598 |
|  | MCI Aβ+ diagnosis | –0.41 [–0.80; –0.02] | 0.20 | –2.08 | 283.30 | 0.02 | .038* |
|  | DAT Aβ+ diagnosis | –0.90 [–1.39; –0.41] | 0.25 | –3.59 | 283.93 | 0.04 | < .001*** |
|  | Age, years ⨉ years since baseline | 0.00 [0.00; 0.00] | 0.00 | –0.72 | 250.62 | 0.00 | .469 |
|  | Sex, male ⨉ years since baseline | 0.00 [–0.03; 0.03] | 0.01 | 0.03 | 239.93 | 0.00 | .973 |
|  | HC Aβ+ diagnosis ⨉ years since baseline | 0.01 [–0.04; 0.05] | 0.02 | 0.25 | 221.92 | 0.00 | .804 |
|  | SCD Aβ+ diagnosis ⨉ years since baseline | –0.04 [–0.07; 0.00] | 0.02 | –1.87 | 249.67 | 0.01 | .063 |
|  | MCI Aβ+ diagnosis ⨉ years since baseline | –0.06 [–0.11; –0.02] | 0.02 | –2.67 | 257.60 | 0.03 | .008** |
|  | DAT Aβ+ diagnosis ⨉ years since baseline | –0.16 [–0.22; –0.09] | 0.03 | –4.90 | 282.91 | 0.08 | < .001*** |
| Posterior cingulate cortex, average thickness  *R*^2^_conditional_ = 0.89  *R*^2^_marginal_ = 0.08 | Intercept | 1.52 [0.04; 3.00] | 0.75 | 2.01 | 316.86 | 0.01 | .045* |
|  | Age, years | –0.02 [–0.04; 0.00] | 0.01 | –1.96 | 316.85 | 0.01 | .051 |
|  | Years since baseline | 0.20 [–0.07; 0.47] | 0.14 | 1.45 | 721.93 | 0.00 | .147 |
|  | Sex, male | –0.13 [–0.37; 0.12] | 0.12 | –1.02 | 316.17 | 0.00 | .308 |
|  | HC Aβ+ diagnosis | 0.01 [–0.41; 0.43] | 0.21 | 0.04 | 314.83 | 0.00 | .971 |
|  | SCD Aβ+ diagnosis | –0.11 [–0.44; 0.22] | 0.17 | –0.66 | 316.83 | 0.00 | .507 |
|  | MCI Aβ+ diagnosis | –0.28 [–0.68; 0.13] | 0.21 | –1.34 | 317.07 | 0.01 | .180 |
|  | DAT Aβ+ diagnosis | –0.51 [–1.02; 0.00] | 0.26 | –1.96 | 318.89 | 0.01 | .051 |
|  | Age, years ⨉ years since baseline | 0.00 [–0.01; 0.00] | 0.00 | –1.38 | 722.19 | 0.00 | .169 |
|  | Sex, male ⨉ years since baseline | –0.02 [–0.06; 0.02] | 0.02 | –1.01 | 720.27 | 0.00 | .315 |
|  | HC Aβ+ diagnosis ⨉ years since baseline | 0.02 [–0.05; 0.09] | 0.04 | 0.52 | 718.77 | 0.00 | .601 |
|  | SCD Aβ+ diagnosis ⨉ years since baseline | –0.02 [–0.08; 0.04] | 0.03 | –0.69 | 721.56 | 0.00 | .490 |
|  | MCI Aβ+ diagnosis ⨉ years since baseline | –0.05 [–0.13; 0.02] | 0.04 | –1.40 | 721.81 | 0.00 | .162 |
|  | DAT Aβ+ diagnosis ⨉ years since baseline | –0.25 [–0.35; –0.15] | 0.05 | –4.92 | 724.29 | 0.03 | < .001*** |
| Precuneus, average thickness *R*^2^_conditional_ = 0.94  *R*^2^_marginal_ = 0.25 | Intercept | 2.67 [1.22; 4.12] | 0.74 | 3.62 | 283.22 | 0.04 | < .001*** |
|  | Age, years | –0.04 [–0.06; –0.02] | 0.01 | –3.54 | 283.21 | 0.04 | < .001*** |
|  | Years since baseline | –0.04 [–0.33; 0.26] | 0.15 | –0.24 | 265.24 | 0.00 | .814 |
|  | Sex, male | –0.12 [–0.36; 0.12] | 0.12 | –1.01 | 282.65 | 0.00 | .311 |
|  | HC Aβ+ diagnosis | 0.40 [–0.01; 0.81] | 0.21 | 1.92 | 281.63 | 0.01 | .056 |
|  | SCD Aβ+ diagnosis | –0.09 [–0.41; 0.22] | 0.16 | –0.58 | 283.18 | 0.00 | .564 |
|  | MCI Aβ+ diagnosis | –0.62 [–1.02; –0.23] | 0.20 | –3.10 | 283.37 | 0.03 | .002** |
|  | DAT Aβ+ diagnosis | –1.38 [–1.88; –0.88] | 0.26 | –5.39 | 284.78 | 0.09 | < .001*** |
|  | Age, years ⨉ years since baseline | 0.00 [0.00; 0.00] | 0.00 | –0.09 | 264.58 | 0.00 | .925 |
|  | Sex, male ⨉ years since baseline | –0.04 [–0.09; 0.01] | 0.02 | –1.69 | 252.59 | 0.01 | .092 |
|  | HC Aβ+ diagnosis ⨉ years since baseline | –0.02 [–0.10; 0.06] | 0.04 | –0.48 | 234.62 | 0.00 | .631 |
|  | SCD Aβ+ diagnosis ⨉ years since baseline | –0.10 [–0.17; –0.04] | 0.03 | –3.10 | 262.36 | 0.04 | .002** |
|  | MCI Aβ+ diagnosis ⨉ years since baseline | –0.11 [–0.20; –0.03] | 0.04 | –2.78 | 270.59 | 0.03 | .006** |
|  | DAT Aβ+ diagnosis ⨉ years since baseline | –0.32 [–0.43; –0.22] | 0.06 | –5.89 | 298.62 | 0.10 | < .001*** |
| Infertior parietal cortex, average thickness  *R*^2^_conditional_ = 0.93  *R*^2^_marginal_ = 0.30 | Intercept | 3.86 [2.35; 5.36] | 0.77 | 5.03 | 283.37 | 0.08 | < .001*** |
|  | Age, years | –0.05 [–0.08; –0.03] | 0.01 | –4.87 | 283.36 | 0.08 | < .001*** |
|  | Years since baseline | –0.03 [–0.38; 0.32] | 0.18 | –0.16 | 275.11 | 0.00 | .870 |
|  | Sex, male | –0.28 [–0.53; –0.03] | 0.13 | –2.19 | 282.65 | 0.02 | .029* |
|  | HC Aβ+ diagnosis | 0.07 [–0.36; 0.50] | 0.22 | 0.32 | 281.39 | 0.00 | .749 |
|  | SCD Aβ+ diagnosis | –0.06 [–0.39; 0.27] | 0.17 | –0.36 | 283.31 | 0.00 | .716 |
|  | MCI Aβ+ diagnosis | –0.52 [–0.93; –0.11] | 0.21 | –2.47 | 283.55 | 0.02 | .014* |
|  | DAT Aβ+ diagnosis | –1.51 [–2.03; –0.99] | 0.27 | –5.68 | 285.32 | 0.10 | < .001*** |
|  | Age, years ⨉ years since baseline | 0.00 [–0.01; 0.00] | 0.00 | –0.20 | 274.50 | 0.00 | .844 |
|  | Sex, male ⨉ years since baseline | –0.03 [–0.08; 0.03] | 0.03 | –0.90 | 262.07 | 0.00 | .367 |
|  | HC Aβ+ diagnosis ⨉ years since baseline | 0.00 [–0.10; 0.09] | 0.05 | –0.10 | 243.96 | 0.00 | .919 |
|  | SCD Aβ+ diagnosis ⨉ years since baseline | –0.15 [–0.23; –0.08] | 0.04 | –3.90 | 272.01 | 0.05 | < .001*** |
|  | MCI Aβ+ diagnosis ⨉ years since baseline | –0.16 [–0.25; –0.06] | 0.05 | –3.17 | 280.32 | 0.03 | .002** |
|  | DAT Aβ+ diagnosis ⨉ years since baseline | –0.52 [–0.65; –0.39] | 0.07 | –8.00 | 308.72 | 0.17 | < .001*** |

**p* < .05. ***p* < .01. *** *p* < .001. ^†^Fitted without random slopes due to insufficient variance in random slope estimates.

Abbreviations: AMY, amygdala. BA, Brodmann area. CA, cornu ammonis. CA23DG, cornu ammonis 2, 3, and dentate gyrus. CU, cognitively unimpaired. DAT, dementia of the Alzheimer type. ERC, entorhinal cortex. IPC, inferior parietal cortex. MCI, mild cognitive impairment. PCC, posterior cingulate cortex. PHC, parahippocampal cortex. PRE, precuneus. RSC, retrosplenial cortex. SCD, subjective cognitive decline. SUB, subiculum. TAIL, hippocampal tail.

**Supplementary Table 5** Estimated marginal means of the linear fixed effect of years since baseline on structural MRI markers for each clinical disease stage, derived from linear mixed effects models.

| **Marker** | **Clinical disease stage** | ***b* [95% C.I.]** | ***SE*** | ***t*** | ***df*** | ***η*^2^_partial_** | ***p*** |
| --- | --- | --- | --- | --- | --- | --- | --- |
| Amygdala, volume | CU Aβ– | –0.05 [–0.07; –0.03] | 0.01 | –4.74 | 257.94 | 0.08 | < .001*** |
|  | CU Aβ + | –0.12 [–0.17; –0.08] | 0.02 | –5.46 | 247.84 | 0.11 | < .001*** |
|  | SCD Aβ + | –0.10 [–0.13; –0.07] | 0.01 | –6.58 | 294.48 | 0.13 | < .001*** |
|  | MCI Aβ + | –0.16 [–0.20; –0.12] | 0.02 | –8.67 | 310.15 | 0.20 | < .001*** |
|  | DAT Aβ + | –0.18 [–0.23; –0.14] | 0.03 | –7.30 | 408.62 | 0.12 | < .001*** |
| CA1, volume | CU Aβ– | –0.02 [–0.04; 0.00] | 0.01 | –2.45 | 223.69 | 0.03 | .015* |
|  | CU Aβ + | –0.04 [–0.08; –0.01] | 0.02 | –2.32 | 224.92 | 0.02 | .021* |
|  | SCD Aβ + | –0.04 [–0.06; –0.01] | 0.01 | –2.58 | 258.92 | 0.03 | .010* |
|  | MCI Aβ + | –0.07 [–0.10; –0.03] | 0.02 | –3.36 | 283.99 | 0.04 | < .001*** |
|  | DAT Aβ + | –0.10 [–0.15; –0.04] | 0.03 | –3.49 | 324.38 | 0.04 | < .001*** |
| CA23DG, volume | CU Aβ– | –0.07 [–0.09; –0.05] | 0.01 | –8.67 | 224.76 | 0.25 | < .001*** |
|  | CU Aβ + | –0.08 [–0.11; –0.04] | 0.02 | –4.17 | 226.03 | 0.07 | < .001*** |
|  | SCD Aβ + | –0.10 [–0.13; –0.07] | 0.01 | –7.22 | 260.35 | 0.17 | < .001*** |
|  | MCI Aβ + | –0.12 [–0.16; –0.08] | 0.02 | –6.17 | 284.96 | 0.12 | < .001*** |
|  | DAT Aβ + | –0.17 [–0.22; –0.12] | 0.03 | –6.27 | 325.70 | 0.11 | < .001*** |
| Subiculum, volume | CU Aβ– | –0.05 [–0.06; –0.03] | 0.01 | –5.71 | 227.59 | 0.13 | < .001*** |
|  | CU Aβ + | –0.06 [–0.10; –0.03] | 0.02 | –3.36 | 229.01 | 0.05 | < .001*** |
|  | SCD Aβ + | –0.10 [–0.13; –0.07] | 0.01 | –6.77 | 263.99 | 0.15 | < .001*** |
|  | MCI Aβ + | –0.12 [–0.16; –0.08] | 0.02 | –5.86 | 287.17 | 0.11 | < .001*** |
|  | DAT Aβ + | –0.16 [–0.22; –0.11] | 0.03 | –5.86 | 328.03 | 0.09 | < .001*** |
| Hippocampal tail, volume | CU Aβ– | –0.03 [–0.05; –0.01] | 0.01 | –2.83 | 225.33 | 0.03 | .005** |
|  | CU Aβ + | –0.04 [–0.09; 0.00] | 0.02 | –1.82 | 226.62 | 0.01 | .070 |
|  | SCD Aβ + | –0.08 [–0.12; –0.04] | 0.02 | –4.15 | 261.11 | 0.06 | < .001*** |
|  | MCI Aβ + | –0.06 [–0.11; –0.01] | 0.03 | –2.42 | 285.47 | 0.02 | .016* |
|  | DAT Aβ + | –0.07 [–0.14; 0.00] | 0.04 | –2.00 | 326.34 | 0.01 | .047* |
| Entorhinal cortex, volume | CU Aβ– | –0.01 [–0.03; 0.00] | 0.01 | –1.44 | 225.36 | 0.01 | .150 |
|  | CU Aβ + | –0.08 [–0.13; –0.04] | 0.02 | –3.97 | 226.65 | 0.06 | < .001*** |
|  | SCD Aβ + | –0.06 [–0.09; –0.03] | 0.02 | –3.70 | 261.14 | 0.05 | < .001*** |
|  | MCI Aβ + | –0.09 [–0.14; –0.05] | 0.02 | –4.22 | 285.48 | 0.06 | < .001*** |
|  | DAT Aβ + | –0.19 [–0.25; –0.12] | 0.03 | –5.94 | 326.35 | 0.10 | < .001*** |
| BA35, volume | CU Aβ– | –0.03 [–0.05; –0.01] | 0.01 | –3.36 | 228.50 | 0.05 | < .001*** |
|  | CU Aβ + | –0.10 [–0.15; –0.06] | 0.02 | –4.51 | 230.08 | 0.08 | < .001*** |
|  | SCD Aβ + | –0.08 [–0.11; –0.04] | 0.02 | –4.37 | 265.53 | 0.07 | < .001*** |
|  | MCI Aβ + | –0.08 [–0.12; –0.03] | 0.02 | –3.28 | 288.40 | 0.04 | .001** |
|  | DAT Aβ + | –0.19 [–0.25; –0.13] | 0.03 | –5.74 | 329.24 | 0.09 | < .001*** |
| BA36, volume | CU Aβ– | –0.02 [–0.04; –0.01] | 0.01 | –3.60 | 224.66 | 0.05 | < .001*** |
|  | CU Aβ + | –0.05 [–0.08; –0.02] | 0.02 | –3.55 | 225.93 | 0.05 | < .001*** |
|  | SCD Aβ + | –0.06 [–0.08; –0.03] | 0.01 | –4.89 | 260.22 | 0.08 | < .001*** |
|  | MCI Aβ + | –0.07 [–0.10; –0.04] | 0.02 | –4.26 | 284.88 | 0.06 | < .001*** |
|  | DAT Aβ + | –0.20 [–0.25; –0.16] | 0.02 | –8.97 | 325.56 | 0.20 | < .001*** |
| Parahippocampal cortex, volume^†^ | CU Aβ– | –0.06 [–0.08; –0.04] | 0.01 | –6.51 | 218.75 | 0.16 | < .001*** |
|  | CU Aβ + | –0.03 [–0.07; 0.01] | 0.02 | –1.61 | 220.15 | 0.01 | .109 |
|  | SCD Aβ + | –0.07 [–0.10; –0.04] | 0.02 | –4.31 | 252.56 | 0.07 | < .001*** |
|  | MCI Aβ + | –0.09 [–0.14; –0.05] | 0.02 | –4.27 | 279.03 | 0.06 | < .001*** |
|  | DAT Aβ + | –0.20 [–0.26; –0.14] | 0.03 | –6.42 | 314.76 | 0.12 | < .001*** |
| Retrosplenial cortex, average thickness | CU Aβ– | –0.04 [–0.05; –0.02] | 0.01 | –3.76 | 228.92 | 0.06 | < .001*** |
|  | CU Aβ + | –0.03 [–0.07; 0.01] | 0.02 | –1.42 | 223.58 | 0.01 | .157 |
|  | SCD Aβ + | –0.07 [–0.10; –0.04] | 0.02 | –4.42 | 262.97 | 0.07 | < .001*** |
|  | MCI Aβ + | –0.10 [–0.14; –0.06] | 0.02 | –4.58 | 264.02 | 0.07 | < .001*** |
|  | DAT Aβ + | –0.19 [–0.25; –0.13] | 0.03 | –6.32 | 291.34 | 0.12 | < .001*** |
| Posterior cingulate cortex, average thickness | CU Aβ– | –0.01 [–0.04; 0.02] | 0.01 | –0.46 | 717.73 | 0.00 | .647 |
|  | CU Aβ + | 0.01 [–0.05; 0.08] | 0.03 | 0.37 | 718.61 | 0.00 | .710 |
|  | SCD Aβ + | –0.03 [–0.08; 0.02] | 0.03 | –1.09 | 722.76 | 0.00 | .278 |
|  | MCI Aβ + | –0.06 [–0.13; 0.01] | 0.03 | –1.74 | 721.88 | 0.00 | .081 |
|  | DAT Aβ + | –0.26 [–0.35; –0.16] | 0.05 | –5.32 | 724.34 | 0.04 | < .001*** |
| Precuneus, average thickness | CU Aβ– | –0.07 [–0.10; –0.04] | 0.02 | –4.32 | 235.45 | 0.07 | < .001*** |
|  | CU Aβ + | –0.09 [–0.16; –0.02] | 0.04 | –2.47 | 230.97 | 0.03 | .014* |
|  | SCD Aβ + | –0.17 [–0.23; –0.12] | 0.03 | –6.25 | 270.64 | 0.13 | < .001*** |
|  | MCI Aβ + | –0.19 [–0.26; –0.11] | 0.04 | –4.97 | 271.80 | 0.08 | < .001*** |
|  | DAT Aβ + | –0.40 [–0.50; –0.29] | 0.05 | –7.57 | 301.82 | 0.16 | < .001*** |
| Inferior parietal cortex, average thickness | CU Aβ– | –0.08 [–0.12; –0.04] | 0.02 | –4.04 | 236.49 | 0.06 | < .001*** |
|  | CU Aβ + | –0.08 [–0.17; 0.00] | 0.04 | –1.93 | 232.22 | 0.02 | .055 |
|  | SCD Aβ + | –0.23 [–0.30; –0.17] | 0.03 | –7.05 | 271.66 | 0.15 | < .001*** |
|  | MCI Aβ + | –0.23 [–0.32; –0.15] | 0.04 | –5.29 | 272.78 | 0.09 | < .001*** |
|  | DAT Aβ + | –0.60 [–0.72; –0.48] | 0.06 | –9.72 | 302.80 | 0.24 | < .001*** |

**p* < .05. ***p* < .01. *** *p* < .001. ^†^Fitted without random slopes due to insufficient variance in random slope estimates.

Abbreviations: AMY, amygdala. BA, Brodmann area. CA, cornu ammonis. CA23DG, cornu ammonis 2, 3, and dentate gyrus. CU, cognitively unimpaired. DAT, dementia of the Alzheimer type. ERC, entorhinal cortex. IPC, inferior parietal cortex. MCI, mild cognitive impairment. PCC, posterior cingulate cortex. PHC, parahippocampal cortex. PRE, precuneus. RSC, retrosplenial cortex. SCD, subjective cognitive decline. SUB, subiculum. TAIL, hippocampal tail.

**Supplementary Table 6** Pairwise contrasts of clinical disease stages for estimated marginal means of the linear fixed effect of years since baseline on atrophy markers, derived from linear mixed-effects models.

| **Marker** | **Contrast** | ***b* [95% C.I.]** | ***SE*** | ***t*** | ***df*** | ***η*^2^_partial_** | ***p*** | ***p*_FDR_** |
| --- | --- | --- | --- | --- | --- | --- | --- | --- |
| Amygdala, volume | CU Aβ+ *versus* CU Aβ – | –0.07 [–0.12; –0.03] | 0.02 | –2.99 | 248.12 | 0.03 | .003** | .007** |
|  | SCD Aβ + *versus* CU Aβ – | –0.05 [–0.09; –0.01] | 0.02 | –2.69 | 279.61 | 0.03 | .007** | .012* |
|  | MCI Aβ + *versus* CU Aβ – | –0.11 [–0.16; –0.07] | 0.02 | –5.18 | 298.31 | 0.08 | < .001*** | < .001*** |
|  | DAT Aβ + *versus* CU Aβ – | –0.14 [–0.19; –0.08] | 0.03 | –4.89 | 375.01 | 0.06 | < .001*** | < .001*** |
|  | SCD Aβ + *versus* CU Aβ + | 0.02 [–0.03; 0.08] | 0.03 | 0.88 | 261.33 | 0.00 | .379 | .442 |
|  | MCI Aβ + *versus* SCD Aβ + | –0.06 [–0.11; –0.02] | 0.02 | –2.66 | 304.10 | 0.02 | .008** | .012* |
|  | DAT Aβ + *versus* MCI Aβ + | –0.02 [–0.08; 0.04] | 0.03 | –0.75 | 374.25 | 0.00 | .451 | .451 |
| CA1, volume | CU Aβ+ *versus* CU Aβ – | –0.02 [–0.06; 0.02] | 0.02 | –1.11 | 223.84 | 0.01 | .269 | .425 |
|  | SCD Aβ + *versus* CU Aβ – | –0.02 [–0.05; 0.02] | 0.02 | –0.98 | 246.34 | 0.00 | .326 | .425 |
|  | MCI Aβ + *versus* CU Aβ – | –0.05 [–0.09; 0.00] | 0.02 | –2.13 | 273.69 | 0.02 | .034* | .120 |
|  | DAT Aβ + *versus* CU Aβ – | –0.08 [–0.13; –0.02] | 0.03 | –2.62 | 313.32 | 0.02 | .009** | .064 |
|  | SCD Aβ + *versus* CU Aβ + | 0.01 [–0.04; 0.05] | 0.02 | 0.26 | 237.49 | 0.00 | .795 | .795 |
|  | MCI Aβ + *versus* SCD Aβ + | –0.03 [–0.08; 0.02] | 0.02 | –1.22 | 277.33 | 0.01 | .225 | .425 |
|  | DAT Aβ + *versus* MCI Aβ + | –0.03 [–0.10; 0.04] | 0.03 | –0.91 | 309.03 | 0.00 | .365 | .425 |
| CA23DG, volume | CU Aβ+ *versus* CU Aβ – | –0.01 [–0.04; 0.03] | 0.02 | –0.28 | 224.97 | 0.00 | .783 | .783 |
|  | SCD Aβ + *versus* CU Aβ – | –0.03 [–0.06; 0.00] | 0.02 | –1.86 | 247.61 | 0.01 | .064 | .150 |
|  | MCI Aβ + *versus* CU Aβ – | –0.05 [–0.09; –0.01] | 0.02 | –2.30 | 274.61 | 0.02 | .022* | .079 |
|  | DAT Aβ + *versus* CU Aβ – | –0.10 [–0.16; –0.04] | 0.03 | –3.49 | 314.54 | 0.04 | < .001*** | .004** |
|  | SCD Aβ + *versus* CU Aβ + | –0.03 [–0.07; 0.02] | 0.02 | –1.11 | 238.69 | 0.01 | .268 | .376 |
|  | MCI Aβ + *versus* SCD Aβ + | –0.02 [–0.06; 0.03] | 0.02 | –0.75 | 278.51 | 0.00 | .453 | .528 |
|  | DAT Aβ + *versus* MCI Aβ + | –0.05 [–0.11; 0.01] | 0.03 | –1.55 | 310.25 | 0.01 | .122 | .214 |
| Subiculum, volume | CU Aβ+ *versus* CU Aβ – | –0.02 [–0.06; 0.03] | 0.02 | –0.73 | 227.97 | 0.00 | .463 | .463 |
|  | SCD Aβ + *versus* CU Aβ – | –0.05 [–0.08; –0.02] | 0.02 | –2.91 | 250.87 | 0.03 | .004** | .009** |
|  | MCI Aβ + *versus* CU Aβ – | –0.07 [–0.11; –0.03] | 0.02 | –3.13 | 276.79 | 0.03 | .002** | .007** |
|  | DAT Aβ + *versus* CU Aβ – | –0.12 [–0.17; –0.06] | 0.03 | –3.92 | 316.77 | 0.05 | < .001*** | < .001*** |
|  | SCD Aβ + *versus* CU Aβ + | –0.03 [–0.08; 0.01] | 0.02 | –1.46 | 241.86 | 0.01 | .145 | .237 |
|  | MCI Aβ + *versus* SCD Aβ + | –0.02 [–0.07; 0.03] | 0.02 | –0.75 | 281.32 | 0.00 | .452 | .463 |
|  | DAT Aβ + *versus* MCI Aβ + | –0.05 [–0.11; 0.02] | 0.03 | –1.38 | 312.60 | 0.01 | .169 | .237 |
| Hippocampal tail, volume | CU Aβ+ *versus* CU Aβ – | –0.01 [–0.07; 0.04] | 0.03 | –0.50 | 225.56 | 0.00 | .614 | .734 |
|  | SCD Aβ + *versus* CU Aβ – | –0.05 [–0.09; 0.00] | 0.02 | –2.12 | 248.28 | 0.02 | .035* | .247 |
|  | MCI Aβ + *versus* CU Aβ – | –0.03 [–0.09; 0.02] | 0.03 | –1.12 | 275.09 | 0.00 | .263 | .484 |
|  | DAT Aβ + *versus* CU Aβ – | –0.04 [–0.12; 0.03] | 0.04 | –1.09 | 315.14 | 0.00 | .277 | .484 |
|  | SCD Aβ + *versus* CU Aβ + | –0.03 [–0.09; 0.03] | 0.03 | –1.09 | 239.33 | 0.00 | .275 | .484 |
|  | MCI Aβ + *versus* SCD Aβ + | 0.02 [–0.05; 0.08] | 0.03 | 0.48 | 279.13 | 0.00 | .629 | .734 |
|  | DAT Aβ + *versus* MCI Aβ + | –0.01 [–0.10; 0.08] | 0.04 | –0.22 | 310.86 | 0.00 | .824 | .824 |
| Entorhinal cortex, volume | CU Aβ+ *versus* CU Aβ – | –0.07 [–0.12; –0.02] | 0.02 | –3.01 | 225.59 | 0.04 | .003** | .007** |
|  | SCD Aβ + *versus* CU Aβ – | –0.05 [–0.08; –0.01] | 0.02 | –2.42 | 248.31 | 0.02 | .016* | .023* |
|  | MCI Aβ + *versus* CU Aβ – | –0.08 [–0.13; –0.03] | 0.02 | –3.29 | 275.10 | 0.04 | .001** | .004** |
|  | DAT Aβ + *versus* CU Aβ – | –0.17 [–0.24; –0.11] | 0.03 | –5.23 | 315.14 | 0.08 | < .001*** | < .001*** |
|  | SCD Aβ + *versus* CU Aβ + | 0.02 [–0.03; 0.08] | 0.03 | 0.90 | 239.35 | 0.00 | .369 | .369 |
|  | MCI Aβ + *versus* SCD Aβ + | –0.03 [–0.09; 0.02] | 0.03 | –1.25 | 279.14 | 0.01 | .211 | .246 |
|  | DAT Aβ + *versus* MCI Aβ + | –0.09 [–0.17; –0.02] | 0.04 | –2.42 | 310.86 | 0.02 | .016* | .023* |
| BA35, volume | CU Aβ+ *versus* CU Aβ – | –0.07 [–0.12; –0.02] | 0.02 | –2.73 | 229.00 | 0.03 | .007** | .016* |
|  | SCD Aβ + *versus* CU Aβ – | –0.04 [–0.08; 0.00] | 0.02 | –2.03 | 252.20 | 0.02 | .043* | .075 |
|  | MCI Aβ + *versus* CU Aβ – | –0.04 [–0.09; 0.01] | 0.03 | –1.68 | 277.97 | 0.01 | .093 | .131 |
|  | DAT Aβ + *versus* CU Aβ – | –0.16 [–0.22; –0.09] | 0.03 | –4.48 | 317.93 | 0.06 | < .001*** | < .001*** |
|  | SCD Aβ + *versus* CU Aβ + | 0.03 [–0.03; 0.08] | 0.03 | 0.94 | 243.11 | 0.00 | .349 | .408 |
|  | MCI Aβ + *versus* SCD Aβ + | 0.00 [–0.06; 0.05] | 0.03 | –0.08 | 282.71 | 0.00 | .940 | .940 |
|  | DAT Aβ + *versus* MCI Aβ + | –0.11 [–0.19; –0.03] | 0.04 | –2.80 | 313.81 | 0.02 | .005** | .016* |
| BA36, volume | CU Aβ+ *versus* CU Aβ – | –0.03 [–0.06; 0.00] | 0.02 | –1.76 | 224.86 | 0.01 | .080 | .112 |
|  | SCD Aβ + *versus* CU Aβ – | –0.03 [–0.06; –0.01] | 0.01 | –2.37 | 247.49 | 0.02 | .019* | .033* |
|  | MCI Aβ + *versus* CU Aβ – | –0.04 [–0.08; –0.01] | 0.02 | –2.50 | 274.53 | 0.02 | .013* | .030* |
|  | DAT Aβ + *versus* CU Aβ – | –0.18 [–0.23; –0.13] | 0.02 | –7.50 | 314.41 | 0.15 | < .001*** | < .001*** |
|  | SCD Aβ + *versus* CU Aβ + | 0.00 [–0.04; 0.03] | 0.02 | –0.17 | 238.58 | 0.00 | .864 | .864 |
|  | MCI Aβ + *versus* SCD Aβ + | –0.01 [–0.05; 0.03] | 0.02 | –0.58 | 278.40 | 0.00 | .564 | .658 |
|  | DAT Aβ + *versus* MCI Aβ + | –0.13 [–0.19; –0.08] | 0.03 | –4.90 | 310.12 | 0.07 | < .001*** | < .001*** |
| Parahippocampal cortex, volume^†^ | CU Aβ+ *versus* CU Aβ – | 0.03 [–0.02; 0.07] | 0.02 | 1.17 | 218.87 | 0.01 | .243 | .341 |
|  | SCD Aβ + *versus* CU Aβ – | –0.01 [–0.05; 0.03] | 0.02 | –0.49 | 240.66 | 0.00 | .625 | .625 |
|  | MCI Aβ + *versus* CU Aβ – | –0.03 [–0.08; 0.01] | 0.02 | –1.44 | 269.19 | 0.01 | .151 | .293 |
|  | DAT Aβ + *versus* CU Aβ – | –0.14 [–0.21; –0.08] | 0.03 | –4.31 | 304.65 | 0.06 | < .001*** | < .001*** |
|  | SCD Aβ + *versus* CU Aβ + | –0.04 [–0.09; 0.01] | 0.03 | –1.39 | 232.34 | 0.01 | .167 | .293 |
|  | MCI Aβ + *versus* SCD Aβ + | –0.03 [–0.08; 0.03] | 0.03 | –0.95 | 271.62 | 0.00 | .344 | .401 |
|  | DAT Aβ + *versus* MCI Aβ + | –0.11 [–0.18; –0.03] | 0.04 | –2.82 | 300.99 | 0.03 | .005** | .018* |
| Retrosplenial cortex, average thickness | CU Aβ+ *versus* CU Aβ – | 0.01 [–0.04; 0.05] | 0.02 | 0.25 | 223.17 | 0.00 | .804 | .804 |
|  | SCD Aβ + *versus* CU Aβ – | –0.04 [–0.07; 0.00] | 0.02 | –1.86 | 251.07 | 0.01 | .064 | .111 |
|  | MCI Aβ + *versus* CU Aβ – | –0.06 [–0.11; –0.02] | 0.02 | –2.67 | 259.02 | 0.03 | .008** | .027* |
|  | DAT Aβ + *versus* CU Aβ – | –0.16 [–0.22; –0.09] | 0.03 | –4.89 | 284.46 | 0.08 | < .001*** | < .001*** |
|  | SCD Aβ + *versus* CU Aβ + | –0.04 [–0.09; 0.01] | 0.03 | –1.58 | 238.07 | 0.01 | .115 | .161 |
|  | MCI Aβ + *versus* SCD Aβ + | –0.03 [–0.08; 0.02] | 0.03 | –1.06 | 264.23 | 0.00 | .289 | .337 |
|  | DAT Aβ + *versus* MCI Aβ + | –0.09 [–0.17; –0.02] | 0.04 | –2.54 | 280.32 | 0.02 | .012* | .027* |
| Posterior cingulate cortex, average thickness | CU Aβ+ *versus* CU Aβ – | 0.02 [–0.05; 0.09] | 0.04 | 0.52 | 718.32 | 0.00 | .601 | .601 |
|  | SCD Aβ + *versus* CU Aβ – | –0.02 [–0.08; 0.04] | 0.03 | –0.69 | 721.12 | 0.00 | .490 | .572 |
|  | MCI Aβ + *versus* CU Aβ – | –0.05 [–0.13; 0.02] | 0.04 | –1.40 | 721.37 | 0.00 | .162 | .377 |
|  | DAT Aβ + *versus* CU Aβ – | –0.25 [–0.35; –0.15] | 0.05 | –4.92 | 723.85 | 0.03 | < .001*** | < .001*** |
|  | SCD Aβ + *versus* CU Aβ + | –0.04 [–0.12; 0.04] | 0.04 | –0.97 | 720.35 | 0.00 | .334 | .572 |
|  | MCI Aβ + *versus* SCD Aβ + | –0.03 [–0.11; 0.05] | 0.04 | –0.76 | 722.51 | 0.00 | .445 | .572 |
|  | DAT Aβ + *versus* MCI Aβ + | –0.20 [–0.31; –0.08] | 0.06 | –3.39 | 723.55 | 0.02 | < .001*** | .003** |
| Precuneus, average thickness | CU Aβ+ *versus* CU Aβ – | –0.02 [–0.10; 0.06] | 0.04 | –0.48 | 230.59 | 0.00 | .632 | .737 |
|  | SCD Aβ + *versus* CU Aβ – | –0.10 [–0.17; –0.04] | 0.03 | –3.10 | 258.21 | 0.04 | .002** | .005** |
|  | MCI Aβ + *versus* CU Aβ – | –0.11 [–0.20; –0.03] | 0.04 | –2.77 | 266.38 | 0.03 | .006** | .010* |
|  | DAT Aβ + *versus* CU Aβ – | –0.32 [–0.43; –0.22] | 0.06 | –5.88 | 294.35 | 0.11 | < .001*** | < .001*** |
|  | SCD Aβ + *versus* CU Aβ + | –0.08 [–0.17; 0.01] | 0.05 | –1.83 | 245.29 | 0.01 | .069 | .097 |
|  | MCI Aβ + *versus* SCD Aβ + | –0.01 [–0.10; 0.08] | 0.05 | –0.27 | 272.25 | 0.00 | .788 | .788 |
|  | DAT Aβ + *versus* MCI Aβ + | –0.21 [–0.33; –0.09] | 0.06 | –3.32 | 289.78 | 0.04 | .001** | .004** |
| Inferior parietal cortex, average thickness | CU Aβ+ *versus* CU Aβ – | 0.00 [–0.10; 0.09] | 0.05 | –0.10 | 231.83 | 0.00 | .920 | .959 |
|  | SCD Aβ + *versus* CU Aβ – | –0.15 [–0.23; –0.08] | 0.04 | –3.89 | 259.20 | 0.06 | < .001*** | < .001*** |
|  | MCI Aβ + *versus* CU Aβ – | –0.16 [–0.25; –0.06] | 0.05 | –3.17 | 267.36 | 0.04 | .002** | .003** |
|  | DAT Aβ + *versus* CU Aβ – | –0.52 [–0.65; –0.39] | 0.07 | –7.99 | 295.33 | 0.18 | < .001*** | < .001*** |
|  | SCD Aβ + *versus* CU Aβ + | –0.15 [–0.25; –0.04] | 0.05 | –2.74 | 246.43 | 0.03 | .007** | .009** |
|  | MCI Aβ + *versus* SCD Aβ + | 0.00 [–0.11; 0.10] | 0.05 | –0.05 | 273.29 | 0.00 | .959 | .959 |
|  | DAT Aβ + *versus* MCI Aβ + | –0.37 [–0.51; –0.22] | 0.08 | –4.89 | 290.78 | 0.08 | < .001*** | < .001*** |

**p* < .05. ***p* < .01. *** *p* < .001. ^†^Fitted without random slopes due to insufficient variance in random slope estimates.

Abbreviations: AMY, amygdala. BA, Brodmann area. CA, cornu ammonis. CA23DG, cornu ammonis 2, 3, and dentate gyrus. CU, cognitively unimpaired. DAT, dementia of the Alzheimer type. ERC, entorhinal cortex. IPC, inferior parietal cortex. MCI, mild cognitive impairment. PCC, posterior cingulate cortex. PHC, parahippocampal cortex. PRE, precuneus. RSC, retrosplenial cortex. SCD, subjective cognitive decline. SUB, subiculum. TAIL, hippocampal tail.
